# Fluorescent ligands for dopamine D_2_/D_3_ receptors

**DOI:** 10.1038/s41598-020-78827-9

**Published:** 2020-12-14

**Authors:** Anni Allikalt, Nirupam Purkayastha, Khajidmaa Flad, Maximilian F. Schmidt, Alina Tabor, Peter Gmeiner, Harald Hübner, Dorothee Weikert

**Affiliations:** Department of Chemistry and Pharmacy, Medicinal Chemistry, Friedrich-Alexander-Universität Erlangen-Nürnberg, Nikolaus-Fiebiger-Str. 10, 91058 Erlangen, Germany

**Keywords:** G protein-coupled receptors, Medicinal chemistry, Chemical tools

## Abstract

Fluorescent ligands are versatile tools for the study of G protein-coupled receptors. Depending on the fluorophore, they can be used for a range of different applications, including fluorescence microscopy and bioluminescence or fluorescence resonance energy transfer (BRET or FRET) assays. Starting from phenylpiperazines and indanylamines, privileged scaffolds for dopamine D_2_-like receptors, we developed dansyl-labeled fluorescent ligands that are well accommodated in the binding pockets of D_2_ and D_3_ receptors. These receptors are the target proteins for the therapy for several neurologic and psychiatric disorders, including Parkinson’s disease and schizophrenia. The dansyl-labeled ligands exhibit binding affinities up to 0.44 nM and 0.29 nM at D_2_R and D_3_R, respectively. When the dansyl label was exchanged for sterically more demanding xanthene or cyanine dyes, fluorescent ligands 10a-c retained excellent binding properties and, as expected from their indanylamine pharmacophore, acted as agonists at D_2_R. While the Cy3B-labeled ligand 10b was used to visualize D_2_R and D_3_R on the surface of living cells by total internal reflection microscopy, ligand 10a comprising a rhodamine label showed excellent properties in a NanoBRET binding assay at D_3_R.

## Introduction

Dopamine receptors belong to the large family of G protein-coupled receptors (GPCRs). With their transmembrane architecture, these proteins are responsible for signal transduction from the extracellular environment to intracellular compartments. Dopamine receptors respond to the binding of the neurotransmitter/hormone dopamine and are divided into two sub-groups, the D_1_-like (D_1_R, D_5_R) and the D_2_-like (D_2_R, D_3_R, D_4_R) family, depending on their G protein coupling preference^1^. D_2_-like receptors represent the main targets for the therapy of severe neurological and psychiatric disorders including Parkinson’s disease, schizophrenia, restless legs syndrome, and addiction^1^. Over the past years, high-resolution X-ray crystal structures or cryo-electron microscopy maps have become available for the entire subfamily (D_2_R: 6LUQ^2^, 6CM4^3^, 6VMS^4^; D_3_R: 3PBL^5^; D_4_R: 6IQL^6^, 5WIV^7^, 5WIU^7^), enabling structure-based drug design^7^ as an alternative to ligand-based drug development. Independent from the employed design strategy, drug discovery campaigns require fast and reliable in vitro assays to determine target affinity, selectivity, kinetics and functional activity of novel lead structures. Whereas numerous assay technologies, such as enzyme fragment complementation, fluorescence or bioluminescence resonance energy transfer (FRET or BRET), proximity-based assays and even label-free methods have been employed for the determination of functional activity at GPCRs^8^, the characterization of a ligand’s affinity towards a given receptor is mostly based on radioligand competition. This binding assay has been proven useful due to the high chemical similarity of unlabeled and labeled probe molecules, the highly specific detection of radioactive labels, and the relatively easy assay format^9^. Nevertheless, radioligand-based methods have several disadvantages. Besides the costs and problems associated with the disposal of radioactive waste and regulatory requirements, high-affinity radioactive probe molecules have to be available for the receptor of interest^9^.

Over recent years, fluorescence-based technologies have emerged as exciting alternative to study ligand affinity^10–12^. Similar to radioactive probes, fluorescent molecules can be detected at very low concentration with high specificity. Although the synthesis of small-molecule fluorescent ligands is far from trivial, it requires lower level regulatory and safety precautions compared to the preparation of radioligands. Moreover, several fluorescence-based technologies like resonance energy transfer (RET) and fluorescence anisotropy (also known as fluorescence polarization) do not require separation of unbound ligands and assays can thus be run in a homogenous, fast “mix-and-measure” setup^10,13^. In combination with sophisticated techniques like total internal reflection fluorescence (TIRF) microscopy, fluorescent ligands allow to detect and track GPCRs with single molecule resolution^14–16^. Fluorescent ligands have also been used to monitor receptor internalization^17^, and to study receptor oligomerization in native tissue^18^. Very recently, the development of a small and bright luciferase variant (nanoluciferase, Nluc)^19^ has greatly facilitated the implementation of BRET-based ligand binding assays^11^. In contrast to *Renilla* luciferase, which is frequently used to study intracellular protein–protein interactions, N-terminal Nluc fusion proteins can be easily targeted to the cell surface^11^. NanoBRET between fluorescently labeled ligands and Nluc-receptor fusion proteins has already been used to study ligand binding at a number of therapeutically relevant GRCRs, including β-adrenergic^11^, adenosine^11^, histamine^20^, free fatty acid^21^, and chemokine^22^ receptors. Despite the high therapeutic relevance of the D_2_-like receptor family, only a small number of fluorescent ligands targeting these receptors have been reported so far. Whereas a fluorescently labeled agonist (2-(*N*-phenethyl-*N*-propyl)amino-5-hydroxytetralin scaffold, PPHT-red)^23,24^ has been employed in FRET ligand binding studies at D_2_R (Tag-lite, Cisbio), NanoBRET binding assays have not yet been established for this receptor family. Besides the agonistic PPHT derivatives, different fluorescent ligands comprising an antagonistic *N*-(*p*-aminophenethyl)spiperone (NAPS) scaffold have also been described^25–28^. For example, a Cy3B-labeled NAPS derivative has been used for microscopic analyses of ligand binding at D_3_R^29^. In our group, we have developed Cy3B-labeled fluorescent ligands with an *N*-propylamino-5-hydroxytetraline or phenylpiperazine substructure for TIRF microscopy studies of D_2_R and D_3_R homodimerization in living cells^14^.

Taking advantage of this expertise, we describe the design, molecular docking, chemical synthesis and pharmacological characterization of novel fluorescent ligands targeting the D_2_-like receptor subfamily. Starting from well-known dopamine receptor recognition elements, we developed dansyl-labeled fluorescent ligands that possess excellent binding affinity at D_2_R and D_3_R receptors. We demonstrate that our design strategy is suitable for accommodating larger xanthene and cyanine fluorophores, while maintaining high-affinity dopamine receptor binding. Depending on the fluorophore, our novel ligands can be used to label D_2_R and D_3_R in living cells (TIRF microscopy) or in a newly established D_3_R-NanoBRET ligand binding assay that will open up the path for future drug discovery campaigns.

## Results

### Ligand design and synthesis

**Figure 1:**
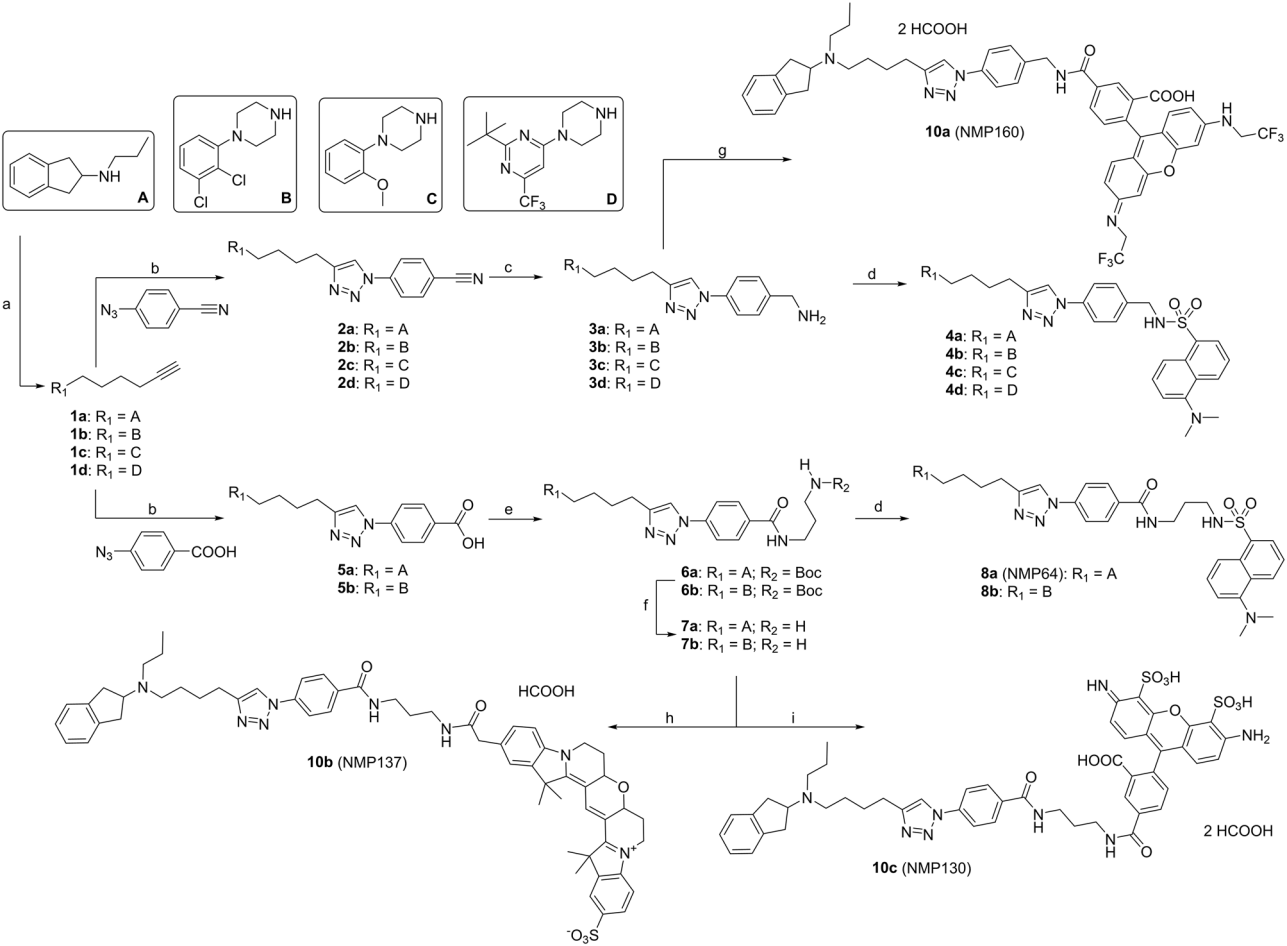
Synthesis of fluorescent ligands **4a-d**, **8a,b** and **10a-c**. Reagents and conditions: (**a**) 6-chlorohex-1-yne, K_2_CO_3_, KI, CH_3_CN, reflux, 68–85%; (**b**) 4-azido-benzonitrile or 4-azidobenzoic acid, 5 mol% CuSO_4_·5H_2_O, 10 mol % Na-ascorbate, 1:1:1 mixture of *tert.*-butanol:H_2_O:CH_2_Cl_2_, rt, 76–92%; (**c**) LiAlH_4_, THF, 0 °C to rt, crude; (**d**) dansyl chloride, triethylamine, CH_2_Cl_2_; 0 °C, 53–88%; (**e**) *tert.*-butyl (3-aminopropyl)carbamate, DIPEA, TBTU, DMF/CH_2_Cl_2_, 0 °C, 85–88%; (**f**) 4 M HCl, dioxane, rt, 98%, (**g**) 5′-carboxy-*N*,*N*′-bis(2,2,2-trifluoroethyl)rhodamine (**9**)^38^, DIPEA, TBTU, CH_2_Cl_2_/DMF, 0 °C, 24 h, 23%; (**h**) Cy3B NHS ester, DIPEA, DMF, rt, 24 h, 66%; (**i**) Alexa488 TFP ester, DIPEA, DMF, rt, 24 h, 58%.

For the development of fluorescent probes targeting the D_2_-like receptor family, we made use of four different privileged scaffolds that are frequently found in dopamine receptor ligands^30^. The *N*-propyl substituted 2-amino-dihydro-1*H*-indene (building block A, Fig. 1), is a well-known dopamine-isostere and exhibits agonistic properties^31,32^. In contrast, 1,4-disubstituted phenyl- or pyrimidyl-piperazines (1,4-DAPs, building blocks B–D, Fig. 1) with different substituents attached to the aromatic core represent the main receptor recognition element of atypical antipsychotics such as aripiprazole and cariprazine^30^. While the 2-methoxy or 2,3-dichloro substituted phenylpiperazines are known to bind to all D_2_-like receptor subtypes, the pyrimidylpiperazine (building block D) selectively targets D_3_R^33^. Previous studies have revealed the importance of a second lipophilic moiety for high-affinity binding to dopamine receptors^30,34^. Thus, we envisioned connection of the primary pharmacophores to a triazole-benzylamine or triazole-benzoic acid moiety through a flexible four-carbon aliphatic linker. This linker size was chosen so that ligands should possess high affinity for both D_2_R and D_3_R, while a considerably shorter linker would have been beneficial for the D_4_R subtype^30^. For an initial set of fluorescent ligands, we focused on the incorporation of a dansyl label, a naphthalene-derived fluorophore emitting green light. The dansyl fluorophore has a relatively low molecular weight and is commercially available as reactive sulfonyl chloride (dansyl chloride). The dansyl dye is widely used to label proteins for fluorescence polarization measurements due to its favorable lifetime or as an acceptor for resonance energy transfer from protein tryptophan residues. It is also known for its large Stokes shift, resulting in a good signal-to-noise ratio^35^. Since we have previously found that the binding pockets of D_2_R and D_3_R can accommodate flexible linkers^14^ but also sterically more demanding substituents in their extended binding pocket^36,37^, installation of the dansyl fluorophore was envisioned through sulfonamide-formation either directly with the primary benzylamine (ligands **4a**-**d**) or through addition of a short 1,3-diaminopropane spacer to the benzoic acid precursors (ligands **8a**,**b**). The latter strategy has already been successful for the development of Cy3B-labeled dopamine receptor ligands in the context of TIRF microscopy^14,17^.

Synthesis of the dansyl-labeled ligands started from the secondary amines *N*-propyl-2,3-dihydro-1*H*-inden-2-amine, 2,3-dichlorophenylpiperazine, 2-methoxyphenylpiperazine or 2-(*tert*-butyl)-4-(piperazin-1-yl)-6-(trifluoromethyl)pyrimidine (Fig. 1, building blocks A–D), that were reacted with 6-chlorohex-1-yne in a nucleophilic displacement reaction in analogy to previously described protocols^39,40^. The resulting terminal alkynes **1a**-**d** were subjected to a copper(I)-catalyzed azide-alkyne cycloaddition with 4-azido-benzonitrile^14,41^, giving access to the 1,4-disubstituted triazoles **2a**-**d** in excellent yield. Subsequent reduction of the benzonitriles with LiAlH_4_ afforded the primary amines **3a**-**d**, which were used in the next step without further purification. In the final step, dansyl-labeled title compounds **4a**-**d** were obtained by reaction with dansyl chloride in presence of triethylamine. Fluorescent ligands **8a**,**b** comprising an additional aminopropane spacer were prepared in a very similar reaction sequence. To this end, the terminal alkynes **1a**,**b** were first reacted with 4-azido-benzoic acid^42^ in a copper(I)-catalyzed azide-alkyne cycloaddition^14,41^ yielding the triazoles **5a**,**b**. The benzoic acid moiety was then coupled to mono *Boc*-protected 1,3-diaminopropane^43^ using TBTU as the coupling reagent, yielding intermediates **6a**,**b**. *Boc*-deprotection was carried out using hydrochloric acid in dioxane, before the resulting primary amines **7a**,**b** were reacted with dansyl chloride to afford the title compounds **8a** and **8b**.

Despite the advantages of the dansyl label, it is not ideally suited for all popular fluorescence-based technologies, for example bioluminescence resonance energy transfer. For instance, its fluorescence properties are highly sensitive to solvent polarity and the fluorescence excitation maximum (λ_max_ ≈ 350 nm)^35^ prevents usage in NanoBRET assays. In recent years, many organic fluorophores with emission maxima in the red spectral range and enhanced photochemical properties have been developed. Chemically, these fluorophores often belong to the xanthene or cyanine dye families, but differ in their substituents, leading to a variety of net charges and different degrees of hydrophilicity^44^. We selected three different fluorophores, the commercially available dyes Alexa488 and Cy3B and a recently described bis-trifluoroethyl substituted rhodamine^38^ for the synthesis of our fluorescent dopamine receptor probes. Alexa488 and Cy3B, but also tetramethylrhodamine dyes (TAMRA), have already been shown to be suitable for characterizing ligand binding to GPCRs^11,22,29,45^, and especially Cy3B-labeled ligands have proven useful for fluorescence microscopy^14,17,29^. For the synthesis of these ligands, we focused on the *N*-propyl substituted 2-amino-dihydro-1*H*-indene scaffold. 5 ´-Carboxy-*N*,*N* ´-bis(2,2,2-trifluoroethyl)rhodamine (**9**) was prepared as described previously^38^ and connected directly to the benzylamine **3a** using TBTU as the coupling reagent to give the desired fluorescent ligand **10a** in a yield of 28%. Alexa488 and Cy3B were purchased as tetrafluorophenyl (TFP) or *N*-hydroxysuccinimid (NHS) esters, respectively, and reacted with the 1,3-dipropylamino-substituted benzoic acid **7a** under basic conditions in DMF, affording the fluorescent ligands **10b** (Cy3B) and **10c** (Alexa488).

### Molecular docking

**Figure 2:**
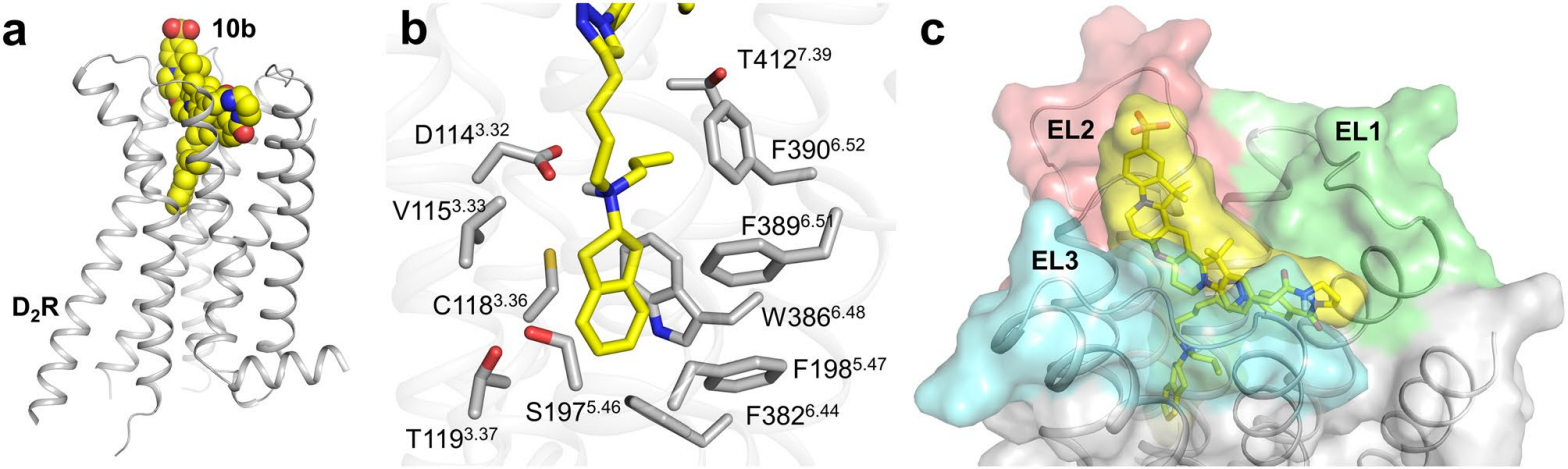
Docking pose of **10b** at D_2_R. (**a**) Overview of the **10b** pose in the D_2_R. (**b**) The stick representation of the indanylamine pharmacophore in the orthosteric binding pocket shows an ionic interaction of the protonated nitrogen with D114^3.32^, while the aromatic ringsystem is accommodated in a hydrophobic pocket formed by V115^3.33^, C118^3.36^, T119^3.37^, S197^5.46^, F198^5.47^, F382^6.44^, W386^6.48^ and F389^6.51^. The propyl substituent of the protonated amine points into a hydrophobic cleft formed by W386^6.48^, F390^6.52^ and T412^7.39^. (**c**) Surface representation of the extracellular loop region. **10b** is colored yellow, while EL1, EL2 and EL3 are colored in green, red and cyan, respectively. The polar sulfonate group of **10b** is directed outwards towards the solvent.

To explore whether addition of the fluorescent moieties would be tolerated by the dopamine receptors and to explore their location upon binding of the probes to D_2_R and D_3_R, docking studies were performed with the representative ligands **8a** and **10b**, comprising a dansyl and a Cy3B fluorophore, respectively, and the crystal structures of D_2_R (complex with risperidone, PDB-ID: 6CM4^3^) or D_3_R (complex with eticlopride, PDB-ID: 3PBL^5^). As expected from the ligand design, typical receptor-ligand interactions in the orthosteric binding pocket^3,5^ including the salt bridge formation with Asp^3.32^ were observed for the indanylamine moiety of **8a** and **10b** in both receptors (Fig. 2, Supplementary Fig. S1). These results indicate that the presence of the fluorescent moiety does not hinder the pharmacophore from adopting its canonical binding pose. Thus, the addition of long and flexible aliphatic spacers, which were previously employed in fluorescent probes targeting D_2_R or D_3_R^14,17^, is not strictly required. According to the docking results, the fluorescent moieties of both ligands lay between the extracellular loops of the receptors, mainly interacting with hydrophobic amino acid sidechains. Between the two receptor subtypes, binding poses of the ligands only differed marginally (Fig. 2, Supplementary Fig. S1).

### Binding affinity and functional activity

Affinities for the dopamine receptor subtypes D_1_–D_4_ along with the related 5-HT_1A_, 5-HT_2_ and α_1_ receptors were determined by radioligand binding for the synthesized fluorescent ligands and some of the central intermediates (Table 1, Supplementary Table S1). In general, ligand affinities were found to be mostly dependent on the pharmacophore. Ligands comprising an *N*-propyl-2-aminoindane or phenylpiperazine substructure showed *K*_*i*_ values in the low nanomolar range for all D_2_-like receptors, with the highest affinities observed at the D_3_R subtype (0.26–13 nM), while the affinities for the D_1_R were substantially lower (180 – > 10,000 nM). The direct comparison of the dansyl-labeled ligands **4a**-**c** with their benzonitrile precursors **2a**-**c** clearly demonstrates that the insertion of the naphthyl-derived fluorophore is well tolerated by D_2L_R, D_2S_R, D_3_R and D_4_R, as highly similar binding affinities were determined in the presence and absence of the fluorescent moiety. As expected, ligands **2d** and **4d**, containing a pyrimidylpiperazine as primary dopamine receptor recognition element, were found to be selective for the D_3_R subtype. The introduction of the dansyl moiety further increased the D_3_R-selectivity, as it had no major influence on the D_3_R affinity (9.5 vs. 11 nM), but decreased the affinity for the D_2L_R and D_2S_R subtypes by a factor of ~ 20. Since **4d** is also selective for D_3_R over the investigated serotonin and α_1_ receptors, it could potentially be used in fluorescence microscopy for D_3_R localization studies in tissues. Connection of the dansyl label and the lipophilic moiety through a short 1,3-diaminopropylene-spacer did not substantially improve binding affinities at the D_2_R and D_3_R, indicating that this additional spacer is not strictly required for the small dansyl fluorophore. However, with a *K*_*i*_ value of 0.44 nM, ligand **8a** displayed the highest D_2S_R affinity of the entire series. When the small dansyl fluorophore was exchanged for the sterically more demanding xanthene or cyanine dyes, binding affinities slightly decreased for the D_2S_ and D_2L_ receptors (4.8–46 nM). Similar to their dansyl analogs, fluorescent ligands **10a–c** displayed the strongest binding affinity for the D_3_R subtype (0.76–0.97 nM). With the exception of the D_3_R-selective ligands **2d** and **4d**, the series of fluorescent ligands and precursors also showed affinity for 5-HT_1A_R (13–260 nM) and α_1_-AR (1.1–150 nM). This is not unexpected, since the 2-methoxyphenylpiperazine is a common motif in α_1_-AR antagonists^46^. At 5-HT_2_R, observed affinities ranged from 220 to 2,200 nM, only.

**Table 1:** Binding affinities of the test compounds.

	*K*_*i*_ [nM]^a^			
	[^3^H]spiperone			
	hD_2L_R	hD_2S_R	hD_3_R	hD_4_R
**2a**	8.7 ± 2.5 (*4*)	6.5 ± 1.7 (*4)*	0.41 ± 0.09 (*4*)	33 ± 7 (*4*)
**2b**	15 ± 5 (*3*)	6.3 ± 0.2 (*3*)	1.0 ± 0.2 (*3*)	120 ± 61 (*3*)
**2c**	77 ± 17 (*3*)	34 ± 8 (*3*)	13 ± 4 (*3*)	140 ± 10 (*3*)
**2d**	360 ± 60 (*4*)	350 ± 30 (*4*)	9.5 ± 1.9 (*4*)	5,000 ± 300 (*4*)
**4a**	5.2 ± 0.6 (*4*)	1.7 ± 0.3 (*5*)	0.29 ± 0.02 (*5*)	27 ± 3 (*5*)
**4b**	20 ± 8 (*3*)	5.1 ± 1.7 (*3*)	1.9 ± 0.7(*3*)	59 ± 20 (*3*)
**4c**	17 ± 7 (*3*)	8.4 ± 3.4 (*3*)	6.5 ± 2.3 (*3*)	32 ± 6 (*3*)
**4d**	7,600 ± 3,000 (*4*)	6,000 ± 1,400 (*4*)	11 ± 2 (*4*)	> 10,000 (*4*)
**7a**	3.5 ± 0.4 (*8*)	1.8 ± 0.2 (*8*)	0.26 ± 0.03 (*8*)	8.5 ± 0.8 (*8*)
**8a** (NMP64)	1.2 ± 0.2 (*4*)	0.44 ± 0.12 (*4*)	0.32 ± 0.05 (*4*)	10 ± 4 (*4*)
**8b**	13 ± 6 (*4*)	3.6 ± 1.6 (*4*)	1.9 ± 0.9 (*4*)	130 ± 10 (*4*)
**10a** (NMP160)	46 ± 7 (*4*)	21 ± 4 (*4*)	0.97 ± 0.23 (*4*)	56 ± 4 (*4*)
**10b** (NMP137)	10 ± 2 (*5*)	4.8 ± 1.0 (*5*)	0.90 ± 0.26 (*5*)	50 ± 7 (*4*)
**10c** (NMP130)	23 ± 5 (*4*)	15 ± 4 (*4*)	0.76 ± 0.11 (*4*)	47 ± 9 (*4*)

Binding affinities of the test compounds for human D_2L_R, D_2S_R, D_3_R, D_4_R were determined by radioligand competition.

^a^Data represent mean ± SEM of (*n*) individual experiments, each performed in triplicates.

In order to evaluate the impact of the fluorophores on the functional activity of the ligands, we determined their capacity to elicit β-arrestin-2 recruitment to the D_2S_R employing an assay based on enzyme fragment complementation (Pathhunter, DiscoverX). Since the presence of GRK2 is known to be important for a sensitive detection of ligand effects in HEK293 cells, GRK2 was coexpressed together with the D_2S_R fused to the enzyme donor. When the cells were incubated with the fluorescent ligands **8a** and **10a–c**, substantial stimulation of β-arrestin-2 recruitment was observed, confirming the agonistic nature of the *N*-propyl-2-aminoidane dopamine-isostere (Supplementary Fig. S2). In good agreement with its high binding affinity, the dansyl-labeled ligand **8a** acted as a full agonist (E_max_ 98 ± 2%) and displayed a potency (EC_50_ 51 ± 11 nM) that is only slightly lower compared to the reference agonist quinpirole (EC_50_ 20 ± 3 nM, E_max_ 100 ± 1%). Incorporation of the larger trifluoroethyl-rhodamine, Cy3B or Alexa488 fluorophores led to a two- to eightfold reduction in ligand potency alongside with slightly reduced ligand efficacy for the fluorescent agonists **10a** (EC_50_ 410 ± 70 nM, E_max_ 89 ± 3%), **10b** (EC_50_ 410 ± 60 nM, E_max_ 79 ± 3%) and **10c** (EC_50_ 100 ± 20 nM, E_max_ 93 ± 3%), respectively. In agreement with our docking studies, these results indicate that D_2_-like receptors can accommodate the small dansyl- but also larger xanthene or cyanine-derived fluorescent ligands. However, not only the size but also the type of the fluorophore can have an impact on binding affinity, intrinsic activity and potency. This is illustrated in particular by the Cy3B-labeled ligand **10b**, which has threefold higher affinity but fourfold lower potency for β-arrestin-2 recruitment at D_2S_R compared to **10c** that only differs from **10b** in terms of the fluorophore.

### TIRF microscopy

**Figure 3:**
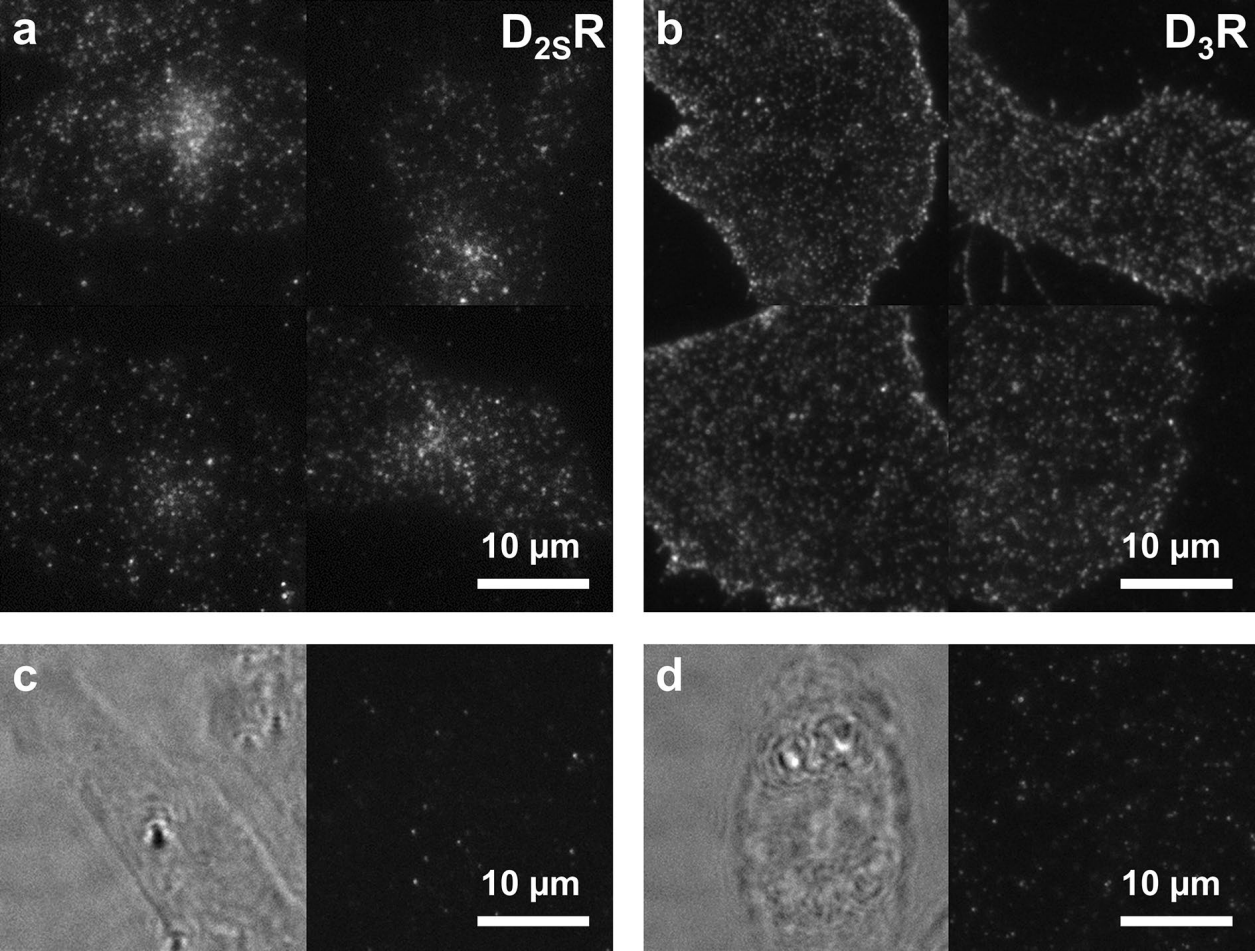
TIRF microscopy imaging with fluorescent ligand **10b**. TIRF images of CHO cells stably expressing (**a**) D_2S_R or (**b**) D_3_R labeled with **10b** (10 nM for D_2S_R, 1 nM for D_3_R) visualize the receptor distribution on the cell surface and demonstrate the suitability of the fluorescent ligand for high resolution fluorescence microscopy. (**c**, **d**) Validation of specific labeling: representative brightfield (left) and TIRF images (right) of a CHO cell stably expressing (**c**) D_2S_R or (**d**) D_3_R, preincubated with the D_2/3_R antagonist spiperone (10 μM) and treated with **10b** (10 nM) show only a few non-specifically adhered, immobile fluorescent spots and no cell-specific labeling.

Fluorescence microscopy, in particular TIRF microscopy, is a powerful method to study the expression, distribution and interactions of GPCRs in the cytoplasmic membrane with high spatial and temporal resolution^14–17,47,48^. We employed our previously developed protocol for the imaging of dopamine receptors^14^ to verify that our newly developed fluorescent ligands are generally suitable for fluorescence imaging. For TIRF microscopy, we focused on the Cy3B-labeled ligand **10b**, as the same fluorophore was previously used for the imaging of D_2_R and D_3_R^14,17^. Indeed, when CHO cells stably expressing D_2S_R or D_3_R were labeled with **10b** in 10 nM (D_2S_R) or 1 nM (D_3_R) concentration, receptors were detected in the cytoplasmic membrane as discrete fluorescent spots (Fig. 3a,b). While D_3_R was found to be evenly distributed over the entire cell membrane, fluorescently labeled D_2S_R showed a more inhomogeneous distribution with clusters of fluorescent puncta. Employing a set of different fluorescently labeled D_2_R ligands, we could previously show that these clusters correspond to internalized receptors^17^. It is not surprising that we could not observe such clusters for D_3_R, because D_3_R is known to hardly undergo agonist-mediated β-arrestin recruitment and internalization^49^. When cells were pretreated with the D_2/3_R antagonist spiperone, neither clustered fluorescent puncta nor labeling of the receptors at the cell surface were observed, indicating that non-specific binding and uptake of ligand **10b** were negligible (Fig. 3c,d).

### NanoBRET

**Figure 4:**
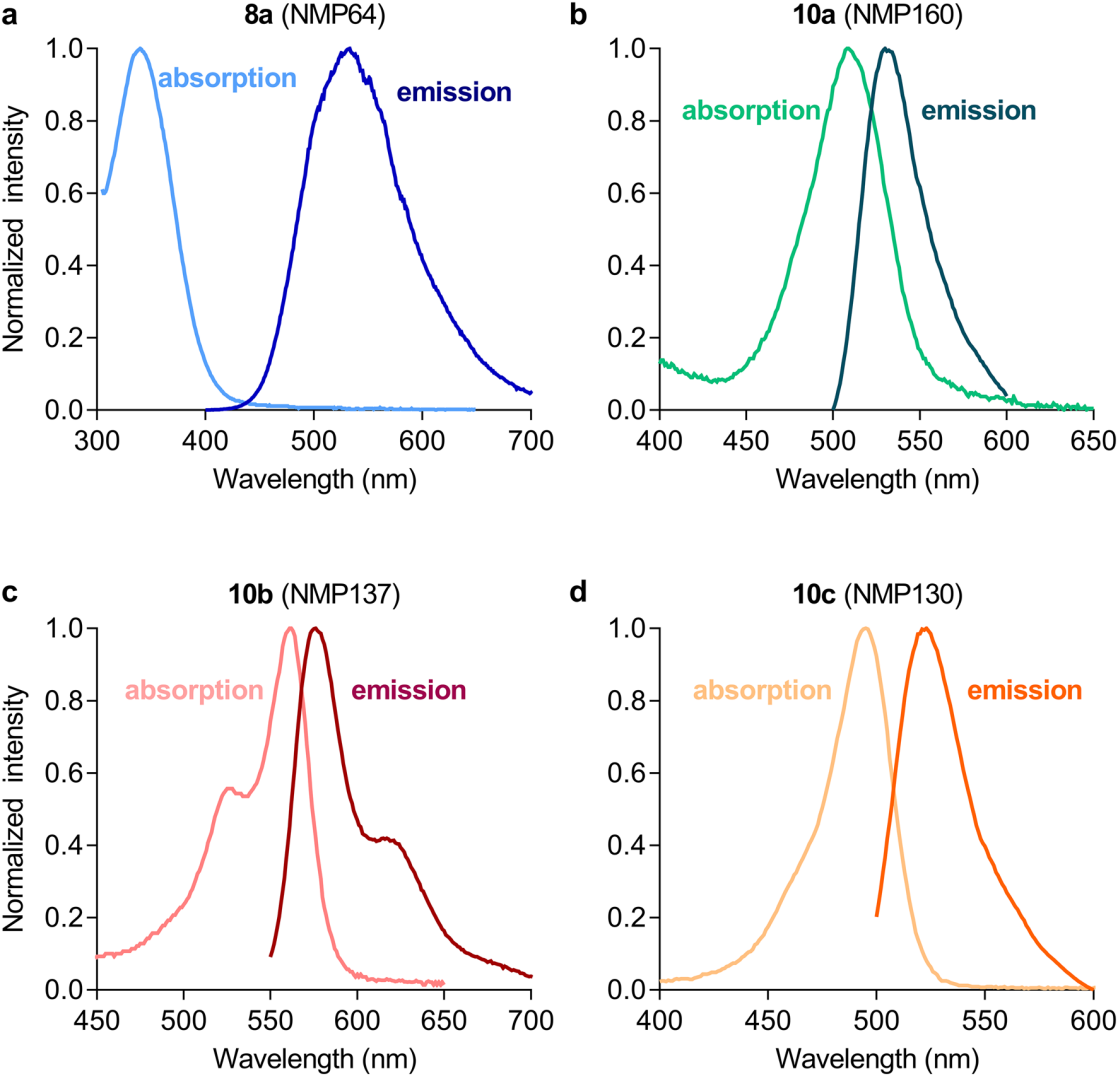
Spectral properties of fluorescent ligands **8a** and **10a-c.** Absorption and fluorescence emission spectra of the fluorescent ligands **8a** and **10a**-**c** were obtained on a microplate reader and normalized to the respective maximum signal of each sample.

To further take advantage of the newly developed ligands as fluorescent probes, we planned to establish a NanoBRET assay for the detection of ligand binding at dopaminergic receptors. In this assay, ligand binding is detected through RET between the bioluminescent Nluc^19^ enzyme fused to the receptor N-terminus and the fluorescent ligand, which only occurs if the two molecules are in sufficient proximity to each other^11^. As a first step, we obtained absorbance and emission spectra of ligands **8a** and **10a**-**c** to identify most suitable candidates. As shown in Fig. 4, ligands **8a**, **10a** and **10c** possess a maximum emission in the range of 540–550 nm, while the emission maximum of the Cy3B fluorophore in **10b** is red-shifted (emission maximum at ~ 600 nm). Analysis of the absorption spectra confirmed the expected spectral properties of the employed fluorophores. While absorption occurred only in the UV range for the dansyl-labeled ligand **8a**, compounds **10a**-**c** showed significant absorption in the area of 450–550 nm (up to 600 nm for **10b**). Thus, ligands **10a**-**c** could serve as BRET acceptors in combination with the Nluc enzyme as BRET donor, which shows luminescence in the range of 400–550 nm (maximum at 460 nm)^19^.

For the development of the assay, we focused on the D_3_R, since our fluorescent ligands showed the highest affinity for this receptor subtype. To this end, a membrane targeted Nluc enzyme^11^ (secNluc, Promega) and D_3_R were fused in frame by polymerase chain reaction and cloned into pcDNA3.1 for mammalian expression. A second construct carrying an N-terminal HA export sequence and a FLAG-tag^50^ in front of the Nluc was generated, which allowed the detection of cell surface expression by ELISA^17^. Upon transient transfection into HEK293T cells, both variants of the Nluc-D_3_R fusion protein were well expressed, as determined by radioligand saturation (B_max_ 2,500–16,200 fmol·mg^−1^ protein for secNluc-D_3_R and 5,000–5,700 fmol·mg^−1^ protein for FLAG-Nluc-D_3_R, compared to 2,200–4,800 fmol·mg^−1^ protein for wild type receptors (wtD_3_R), respectively), and both constructs showed the expected Nluc emission spectra in the presence of the substrate furimazine (Supplementary Fig. S3). A direct comparison of Flag-D_3_R and FLAG-Nluc-D_3_R in an ELISA with an antibody directed against the N-terminal FLAG-tag (Supplementary Fig. S3) showed that the presence of the N-terminal enzyme even improved cell surface expression. On the other hand, the N-terminal Nluc had no influence on the receptor-ligand recognition properties, as binding affinities for the reference ligands haloperidol, cariprazine, aripiprazole and fluspirilene were found to be highly similar to those obtained with wtD_3_R (Supplementary Table S2). In a G protein activation assay (IPOne, Cisbio) with the reference agonist quinpirole, even slightly better potencies were observed for the Nluc-D_3_R fusion constructs compared to unmodified receptors (Supplementary Fig. S3, EC_50_ ± SEM: 14 ± 4 nM for wtD_3_R, 3.7 ± 0.4 nM for secNluc-D_3_R and 4.6 ± 0.8 nM for FLAG-Nluc-D_3_R, respectively), which are likely a result of their higher expression level.

**Figure 5:**
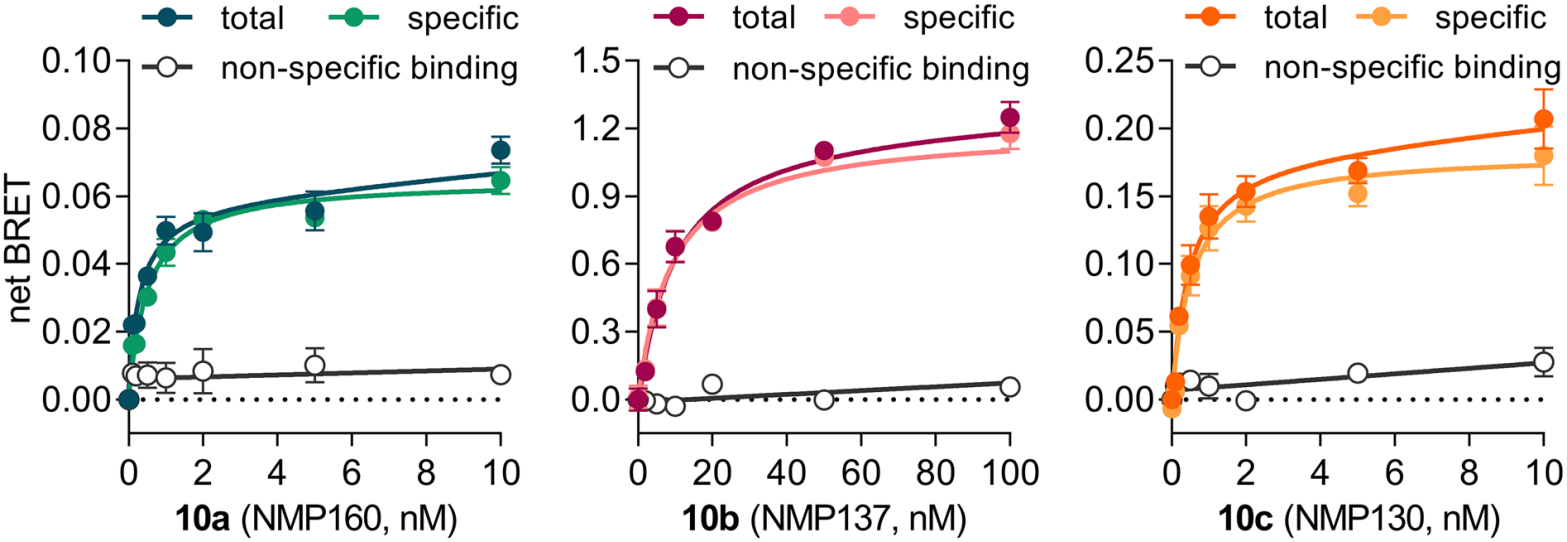
NanoBRET saturation curves for the fluorescent ligands **10a-c**. Saturation binding experiments were performed with live HEK293T cells expressing secNluc-D_3_R and the fluorescent ligands **10a**, **10b** and **10c**, comprising a trifluorethyl-rhodamine derivative, a Cy3B and an Alexa488 fluorophore, respectively. Non-specific binding was determined in the presence of 10 µM haloperidol. Data points show mean ± SEM of one representative out of n = 5 (for **10a**) or n = 4 (for **10b**,**c**) experiments, with each condition carried out in triplicate. The netBRET signal was calculated as the difference between total BRET and the signal obtained in the absence of a fluorescent ligand.

To find out whether fluorescent ligands **10a**-**c** are indeed suitable for BRET-based ligand binding assays, we performed saturation binding assays with live HEK293T cells expressing secNluc-D_3_R. For all three ligands, typical saturation hyperbolas were observed (Fig. 5). Application of 10 µM haloperidol efficiently prevented binding of the fluorescent ligands and demonstrated a low contribution of non-specific binding to the detected netBRET signal. Analysis of the *K*_*D*_ values revealed identical affinities for the trifluoroethyl-rhodamine and Alexa488-labeled ligands **10a** and **10c** (*K*_*D*_ ± SEM 0.72 ± 0.07 nM for **10a**, n = 5; 0.72 ± 0.08 nM, n = 4 for **10c**, respectively), that were in good agreement with their *K*_*i*_ values obtained by radioligand competition (Table 1). In contrast, the determined affinity of the Cy3B-derivative **10b** was substantially lower (*K*_*D*_ ± SEM 12.1 ± 1.7 nM, n = 4), which was surprising, given that its radioligand *K*_*i*_ value (0.90 ± 0.26 nM) was similar to those of **10a** and **10c**. Almost identical results were obtained, when additional saturation experiments with **10a** (*K*_*D*_ ± SEM: 0.90 ± 0.14 nM, n = 5) and **10b** (*K*_*D*_ ± SEM: 11.3 ± 1.6 nM, n = 4) were performed with membranes from HEK293T cells expressing secNluc-D_3_R instead of whole live-cells (Supplementary Fig. S4). This indicates that the higher *K*_*D*_ value of **10b** observed by NanoBRET is not a result from general differences between membrane and whole-cell assays.

**Figure 6:**
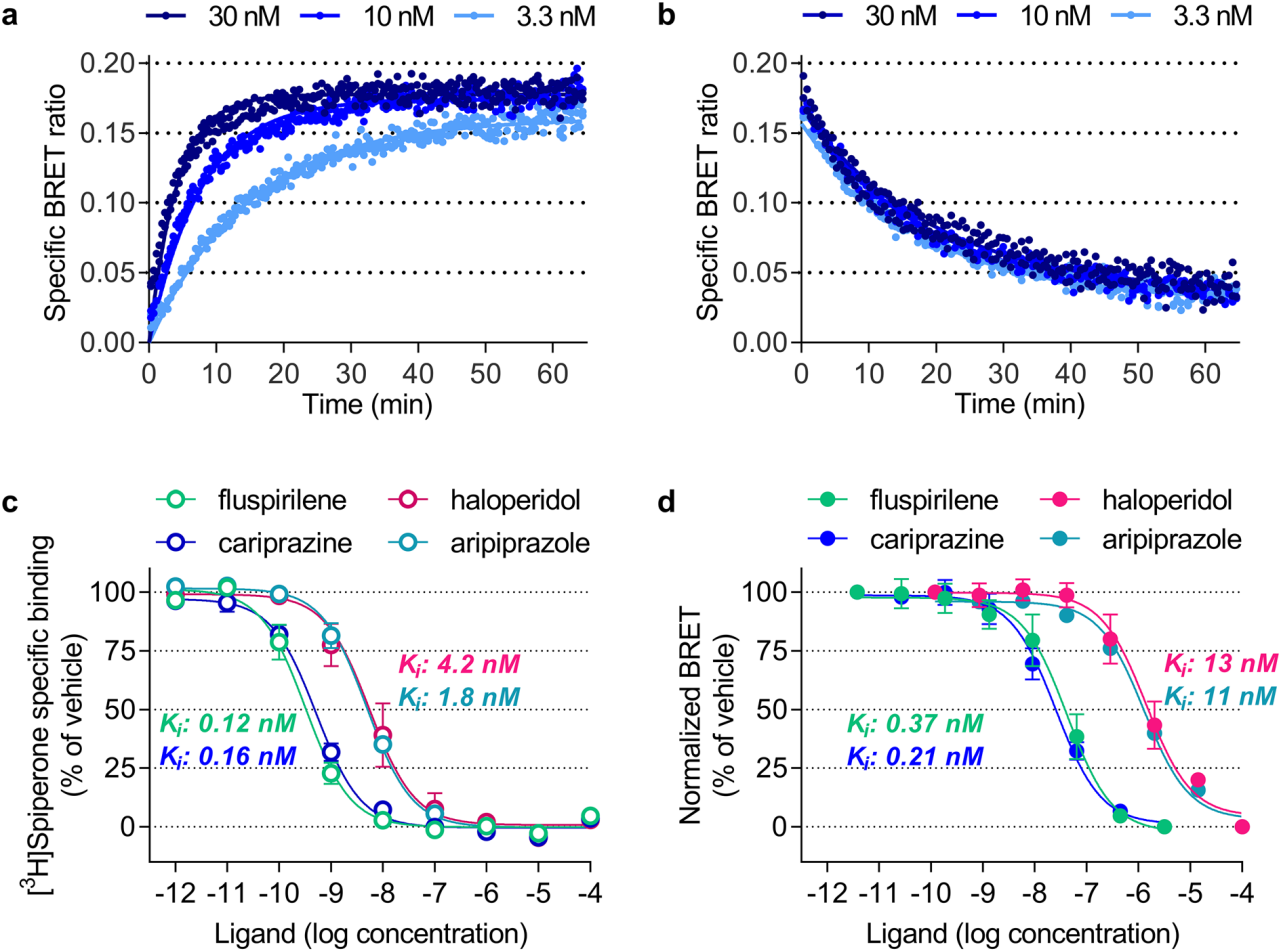
Kinetics of fluorescent ligand 10a at D_3_R and competition binding experiments. (**a**) Association of **10a** at room temperature was measured by NanoBRET using secNluc-D_3_R membrane preparations and threefold serial dilutions of **10a** (3.3–30 nM). (**b**) Dissociation of **10a** was initiated by addition of 10 μM haloperidol after equilibrium had been reached. Data show duplicates of an individual representative experiment. (**c**,**d**) Competition binding curves for reference antipsychotics obtained with (**c**) the radioligand [^3^H]spiperone and membranes from HEK293T cells expressing wtD_3_R receptors or (**d**) 100 nM fluorescent ligand **10a** and secNluc-D_3_R membranes. Obtained IC_50_ values were transformed into *K*_*i*_ values applying the equation of Cheng and Prusoff^52^. Data show mean ± SEM of n = 4 independent experiments.

Association and dissociation experiments performed with fluorescent ligand **10a** at room temperature confirmed its excellent properties and showed a concentration dependent association profile (Fig. 6a), with an association rate constant of 1.02 ± 0.30 × 10^7^ min^−1^ × M^−1^ (*K*_*on*_ ± SEM, n = 10, Supplementary Table S3). As expected, dissociation of **10a** was independent from the employed concentration (Fig. 6b), resulting in a mean residence time of 19 ± 1 min (mean ± SEM, n = 3). This is about 1.5-fold faster than previously observed for the radioligand [^3^H]spiperone at D_3_R^51^.

Taking advantage of the high-affinity binding and the excellent signal to noise ratio of **10a** in the membrane-based BRET assay, we sought to determine the affinity of the antipsychotics haloperidol, fluspirilene, cariprazine and aripirazole for a direct comparison with the data from radioligand binding experiments (Fig. 6c,d). Independent from the employed concentration of **10a** (10 nM or 100 nM), obtained binding affinities of the four antipsychotics showed nearly the same rank order and were only slightly lower (Supplementary Table S4, Fig. 6d) than those obtained by classical radioligand binding (Supplementary Table S2, Fig. 6c). These results demonstrate that **10a** can be successfully employed in membrane-based NanoBRET assays to determine the *K*_*i*_ values of non-labeled ligands.

## Discussion

Fluorescent ligands represent versatile tools for the investigation of diverse biological questions. Similar to radioligands, they can be detected in very low concentration and with high specificity. In the context of GPCR research, fluorescent ligands have been successfully employed to study receptor internalization^17^, receptor-receptor interactions within the cellular membrane^14–16,18^, and recently also ligand binding^11,22–24,29,45,53^, by techniques like fluorescence microscopy, fluorescence polarization and resonance energy transfer. Starting from phenylpiperazine and indanylamine scaffolds, known dopamine-isosteres with antagonistic or agonistic properties, respectively, we have designed and synthesized a small library of fluorescent ligands for D_2_-like receptors. Our initial efforts were directed towards the synthesis of dansyl-labeled probe molecules, as this fluorophore is cost efficient and readily available as reactive sulfonyl chloride. The obtained fluorescent ligands exhibited binding affinities for D_2_R and D_3_R in the subnanomolar range and ligand **8a** also acted as highly potent D_2_R agonist. Despite these favorable characteristics, the application of the ligands is hampered by the fluorescence properties of the dansyl dye, that are in principle amenable to fluorescence microscopy, but not optimally suited for modern RET applications. Encouraged by the binding properties of ligands **4a** and **8a**, we exchanged the dansyl fluorophore by cyanine or xanthene moieties, both of which have been frequently used in fluorescence microscopy or NanoBRET binding assays^11,14,17,22,29,45^. While this exchange had almost no influence for ligand recognition properties at D_3_R, the fluorophore slightly reduced binding affinity at D_2_R.The fluorescent labels also slightly affected the intrinsic activity of the ligands when tested in a β-arrestin-2 recruitment assay at D_2_R. It should be noted that the employed fluorophores not only differ in their molecular weight and steric demand, but also their overall lipophilicity and net charge, which may influence not only receptor recognition and activation, but also the tendency of non-specific binding^44^.

Taking advantage of our newly developed fluorescent ligands, we sought to establish a fluorescent ligand binding assay with the D_3_R subtype serving as an example case. In principle, ligands **10a**-**c** comprising a bis-trifluoroethylrhodamine, a Cy3B or an Alexa488 fluorophores should be well suited for both, FRET and BRET-based technologies. In FRET-based ligand binding assays, the receptor of interest is labeled with a fluorophore, either through the binding of a fluorescently labeled antibody or a self-labeling tag, that can be linked to a small molecule fluorophore^12^. In contrast, NanoBRET binding assays^11^ make use of a small and bright luciferase variant (Nluc)^19^, that is fused to the extracellular part of the investigated GPCR. In both cases, binding of the fluorescent ligand is detected based on RET between the receptor as light emitting donor and the fluorescent ligand serving as the acceptor^10^. Both RET-assays have been proven extremely useful for the characterization of ligand binding at GPCRs, especially if high affinity radioligands are not available or if binding kinetics^10,23,24^ are central to the investigated research question. Typical saturation hyperbolas were obtained for all three ligands when we performed NanoBRET assays with living HEK293T cells expressing an Nluc-D_3_R fusion protein. For two of the ligands, **10a** and **10c**, observed binding affinities were almost identical to those derived from classical radioligand binding studies. This was also true, when NanoBRET experiments were performed with cell membranes instead of whole cells. For the Cy3B-labled ligand **10b**, the NanoBRET *K*_*D*_ was approximately tenfold higher. On the other hand, **10b** showed and excellent behavior in TIRF microscopy studies with CHO cells expressing D_2_R and D_3_R. This illustrates that despite similar absorption and emission spectra, the choice of the fluorophores may be critical not only for ligand binding to the target but has to be tailored to the desired application of the fluorescent ligand.

In summary, we have developed a set of high affinity fluorescent ligands for D_2_R and D_3_R receptors, which are the main targets in the treatment of severe neurological and psychiatric diseases. Depending on the employed fluorophore, the ligands can be used in high-resolution TIRF microscopy or to study ligand binding by resonance energy transfer. The established D_3_R-NanoBRET assay can be equally performed in whole cells and membranes and can be used for ligand binding screenings and characterization of novel ligands for D_3_R in the future.

## Methods

### General procedures (GP) for chemical synthesis

Synthesis of the fluorescent ligands was achieved employing the following general procedures. Detailed protocols and analytical data for the individual compounds are provided as Supplementary Methods and Supplementary Figs. S5 and S6.

#### GP I: Synthesis of terminal alkynes^39,40^

To a mixture of a secondary amine, K_2_CO_3_ (2 eq) and KI (1 eq) in CH_3_CN was added 6-chlorohex-1-yne at room temperature and the reaction mixture was heated to reflux overnight. After addition of H_2_O, the aqueous phase was extracted with CH_2_Cl_2_. The combined organic layers were dried over Na_2_SO_4_ and evaporated under reduced pressure to give the crude product.

#### GP II: Cu(I)-catalyzed 1,3-cycloaddition^41^

To a mixture of corresponding alkynes and aromatic azides in a solvent system of *tert.*-BuOH-H_2_O-CH_2_Cl_2_ (1:1:1) was added CuSO_4_·5H_2_O (5 mol %) and sodium ascorbate (10 mol %) and the suspension was stirred at room temperature. After the completion of the reaction, the suspension was diluted by the addition of H_2_O, extracted with CH_2_Cl_2_, and the combined organic layers were dried over Na_2_SO_4_. After evaporation, the crude residue was purified by silica-gel column chromatography with CH_2_Cl_2_ followed by 95:5 CH_2_Cl_2_-MeOH.

#### GP III: N-dansylation^54^

To a mixture of the crude primary amine and triethylamine (1:1) in CH_2_Cl_2_ was added dansyl chloride at 0 °C. The reaction mixture was stirred overnight at room temperature and the product was extracted with CH_2_Cl_2_. The combined organic layers were dried over Na_2_SO_4_ and concentrated in vacuo to obtain the crude product. Target compounds were isolated by silica-gel column chromatography with CH_2_Cl_2_ followed by 98:2 CH_2_Cl_2_-MeOH.

#### GP IV: Amide coupling

To a solution of the benzoic acid derivatives and DIPEA in CH_2_Cl_2_ at 0 °C was added TBTU in anhydrous DMF and the mixture was stirred for 30 min before a solution of *tert.*-butyl 3-aminopropylcarbamate in CH_2_Cl_2_ was added. After stirring for 2–3 h, saturated NaHCO_3_ solution was added and the mixture was extracted with CH_2_Cl_2_. The combined organic layers were dried over Na_2_SO_4_ and concentrated in vacuo to obtain the crude product. The compounds were isolated by silica-gel column chromatography with 95:5 CH_2_Cl_2_-MeOH.

### Molecular docking

Docking of **8a** and **10b** was performed analogously to previously described protocols^55^. Ligands were geometry optimized by means of Gaussian16^56^ at the B3LYP/6–31 (d,p) level of theory (attributing a formal charge of + 1) and subsequently docked into the crystal structures of the D_2_R (PDB-ID: 6CM4) and the D_3_R (PDB-ID: 3PBL) using AutoDock Vina^57^. We applied a search space of 40 Å × 40 Å × 40 Å due to the large Cy3B moiety of **10b** and to ensure a complete coverage of the binding pocket. Based on the scoring function and experimental data, four ligand-receptor complexes were selected, which were subsequently submitted to energy minimization using the PMEMD module of the AMBER 18 program package^58^. The all-atom force field ff14SB and the general AMBER force field (GAFF) were used for the receptors and ligands, respectively. Parameters for **8a** and **10b** were assigned using antechamber, and charges were calculated using Gaussian16 at the HF/6–31 (d,p) level of theory and the RESP procedure according to the literature^59^. A formal charge of + 1 was defined for the ligands.

### Radioligand competition experiments

Affinities of the fluorescent ligands and precursors towards the human D_2L_R, D_2S_R, D_3_R, D_4_R^60^, and porcine D_1_R, α_1_-AR, 5-HT_1A_R and 5-HT_2_R^61^ were determined as described previously. For D_2L_R, D_2S_R, D_3_R or D_4_R, membranes from CHO cells stably expressing these receptors and the radioligand [^3^H]spiperone were used. Binding studies with D_1_R were carried out with homogenates obtained from porcine striatum and [^3^H]SCH23390. For α_1_R, 5-HT_1A_R and 5-HT_2_R homogenates of porcine cerebral cortex and the radioligands [^3^H]prazosin for α_1_R, [^3^H]WAY100,635 for 5-HT_1A_R, and [^3^H]ketanserin in the presence of 10 µM prazosin for 5-HT_2_R were used. Detailed concentrations, *K*_*D*_ values, etc. are summarized in Supplementary Table S5. Non-specific binding was determined in the presence of 10 µM haloperidol (D_1_R-D_4_R), WAY100,635 (5-HT_1A_R), ketanserin (5-HT_2_R) or prazosin (α_1_R). Data analysis was performed using the algorithms for non-linear regression in PRISM6.0 to provide an IC_50_ value which was transformed into the *K*_*i*_ value employing the equation of Cheng and Prusoff^52^. Radioligand competition with Nluc-D_3_R membranes was performed in an analogous manner.

### TIRF microscopy

TIRF microscopy was performed as previously described^14^. In brief, CHO-K1 cells stably expressing D_2S_R or D_3_R were seeded on 18 mm glass slides coated with fibronectin in phenol red-free DMEM/F12 supplemented with 10% FBS and were allowed to adhere over night at 37 °C, 5% CO_2_. Cells were washed twice with phenol red-free DMEM/F12 containing 10% FBS and incubated with **10b** (1 nM for D_3_R, 10 nM for D_2S_R) for 1 h at 37 °C, before they were washed another three times. Specific labeling was confirmed by preincubation with 10 μM spiperone for 2 h, followed by incubation with the fluorescent ligand **10b** (10 nM). Glass slides were mounted into a custom-built imaging chamber (500 µL) and washed with imaging buffer (137 mM NaCl, 5.4 mM KCl, 2.0 mM CaCl_2_, 1.0 mM MgCl_2_, and 10 mM, HEPES, pH 7.4) twice, before the imaging chamber was placed on the microscope stage. TIRF imaging was performed at 24.0 ± 0.3 °C on a Nikon TI-Eclipse inverted microscope equipped with a 100x, 1.49 NA oil-immersion objective. For excitation, a Nikon D-Eclipse C1 laser box (561 nm), a 561/14 nm excitation filter and a dichroic long-pass mirror (561 nm) were used. Emitted light was passed through an emission filter 609/54 nm (Semrock Rochester) and projected onto a water-cooled EM-CCD camera(Polar Series Accel 250 LC, Thermo Scientific) at -98 °C (512 × 512 FT, DU-897, Andor). To ensure homogenous illumination, only the central quarter of the chip (300 × 300 pixel) was used for analysis. Microscope control and image acquisition were performed with the NIS Elements software (Nikon Instruments).

### Measurement of absorption and emission spectra

Absorption spectra were recorded on a CLARIOstar (BMG Labtech, Ortenberg, Germany) microplate reader. 1 mM solutions of the ligands were prepared in DMSO and measured either directly (**8a**) or after dilution to 10 µM in PBS (**10a**-**c**). Emission spectra were collected employing an excitation wavelength of 335/10, 470/10 or 520/10 nm, respectively. Spectra were background corrected and normalized to the maximum absorbance/emission of each sample.

### cDNA constructs

For the generation of Nluc-D_3_R fusion constructs in pcDNA3.1, sequences of the Nluc enzyme^19^ (pNLF1-N or pNLF1-secN, Promega) and D_3_R (DRD3, cdna.org) were amplified by polymerase chain reaction and fused in frame with a 4 AA linker (GSSG) by Gibson Assembly^62^ (New England Biolabs). To achieve surface expression, the fusion protein was either N-terminally tagged with an HA-signal sequence and a FLAG-tag^50^ or the secretory version of the enzyme^19^ (pNLF1-secN) was used. Sequence integrity was verified by DNA sequencing (Eurofins Genomics).

### Radioligand saturation binding

HEK293T cells were grown to a confluence of 70–80% and transfected with the Nluc-D_3_R plasmids using polyethyleneimine (PEI) as the transfection reagent (PEI/DNA ratio 3:1). After incubation in DMEM/F12 with 10% FBS at 37 °C, 5% CO_2_ for 48 h, cells were harvested and cell membranes were prepared as described previously^34^. The protein concentration was determined using the method of Lowry and bovine serum albumin as standard^63^. Membranes (protein concentration 5–20 µg·mL^−1^) were incubated with the radioligand [^3^H]spiperone (0.05 nM–2.00 nM, Perkin Elmer) for 1 h at 37 °C in binding buffer (50 mM Tris, 1 mM EDTA, 5 mM MgCl_2_, 100 µg·mL^−1^ bacitracin, 5 µg·mL^−1^ soybean trypsin inhibitor, pH 7.4). Non-specific binding was determined in the presence of 10 µM haloperidol. Reactions were terminated by filtration through GF/B filters soaked with 0.3% PEI solution. Dried filters were sealed with scintillation wax and bound radioactivity was determined with a Microbeta Counter (Perkin Elmer). Data were analyzed with the one-site saturation binding model implemented in PRISM8.0 (GraphPad Software Inc., San Diego, CA) to determine the equilibrium dissociation constant (*K*_*D*_) and the receptor expression level (B_max_).

### Live-cell NanoBRET

For whole cell NanoBRET saturation assays, HEK293T cells were transfected with secNluc-D_3_R employing Mirus TransIT-293 (3:1 reagent to DNA ratio). After 24 h at 37 °C, 5% CO_2_, cells were detached using Versene and transferred to white, F-bottom 384-well plates at a density of 2,500 cells/well and incubated for another 24 h at 37 °C, 5% CO_2_. Cells were washed with PBS and incubated for 30 min at 37 °C with 5% FBS in DMEM without phenol red, before the fluorescent ligands (5 µL, diluted into DMEM without phenol red with 5% FBS from a 1 mM DMSO-stock) were added. Non-specific binding was determined in the presence of 10 µM haloperidol. After 1 h incubation with the ligands at 37 °C, furimazine (Promega, final dilution 1:500 to 1:2,500) was added, followed by a 5 min incubation at room temperature in the dark. BRET was measured as the ratio of acceptor fluorescence and donor luminescence employing a CLARIOstar microplate reader equipped with 475/30 nm and 535/30 or 580/30 emission filters, respectively. Total, non-specific and specific binding were analyzed employing the algorithms for one-site saturation binding implemented in PRISM6.0 or 8.0.

### Membrane-based NanoBRET

SecNluc-D_3_R membranes were resuspended in assay buffer^20^ (50 mM Na_2_HPO_4_, 50 mM KH_2_PO_4_, 1 mg·mL^−1^ saponin, 5% FBS, pH 7.4), diluted to a total protein concentration of 0.5–3 μg/well in 384-well plates and incubated with the fluorescent ligands **10a** or **10b** (0–100 nM) at 37 °C for 90 min. Non-specific binding was determined in the presence of 10 μM haloperidol. After addition of furimazine (1:5,000, final volume 35 µL), and 5 min incubation at room temperature in the dark, BRET readings were obtained using a CLARIOstar plate reader equipped with a 475/30 nm donor together with a 535/30 nm (for **10a**) or 620/30 nm (for **10b**) acceptor emission filter. Data were analyzed as described for live-cell NanoBRET. For association kinetics, secNluc-D_3_R membranes were added to various concentrations of **10a** (1–100 nM) in the presence or absence of 10 μM haloperidol and furimazine (1:2,500). For dissociation, membranes were preincubated with **10a** for 1 h, before furimazine was added and dissociation was initiated with 10 μM haloperidol. Kinetic assays were monitored at room temperature and minimal possible cycle time of the CLARIOstar was used for each measurement. Data was fitted to one phase association and dissociation equations in PRISM6.0 to determine the kinetic constants. In competition binding experiments, membranes were incubated with serial dilutions of unlabeled ligands and the fluorescent ligand **10a** (10 or 100 nM) for 90 min at 37 °C. Addition of furimazine (1:5,000) and BRET measurements were carried out as described above. Data analysis was performed using the one site—fit *K*_*i*_ equation in PRISM6.0 to determine the inhibition constants (*K*_*i*_) of the unlabeled ligands.

### Figure preparation

Figures were prepared using ChemDraw, version 18.0.0 (Fig. 1, Perkin Elmer Informatics, www.informatics.perkinelmer.com), PyMOL Molecular Graphics System, version 2.2.1 (Fig. 2, Supplementary Fig. S1, Schrödinger, LLC, www.pymol.org), NIS-Elements AR, version 3.22.13 (Fig. 3, Nikon Instruments, https://www.microscope.healthcare.nikon.com/de_EU/products/software), and GraphPad Prism version 8.0.0 for Windows, (Fig. 4–6, Supplementary Figs. S2–S4, GraphPad Software, San Diego, California USA, www.graphpad.com).

## Supplementary Information

Supplementary Information.

## Data Availability

The data that support the findings of this study are available within the Supplementary Information files and/or from the corresponding authors upon request.
