# Supplementary material for: Fluorescent ligands for dopamine D2/D3 receptors
Source: Sci Rep. 2020 Dec 14;10:21842. doi: 10.1038/s41598-020-78827-9 (PMC7736868; doi:10.1038/s41598-020-78827-9)
Supplement: Supplementary file 1 — Supplementary Information. [file 41598_2020_78827_MOESM1_ESM.pdf]

Supplementary Information (SI)

Fluorescent ligands for dopamine D_2/D_3 receptors

Anni Allikalt[1], Nirupam Purkayastha[1], Khajidmaa Flad[1], Maximilian F. Schmidt[1], Alina Tabor[1], Peter Gmeiner[1], Harald Hübner[1], Dorothee Weikert[1,*]

[1]Department of Chemistry and Pharmacy, Medicinal Chemistry, Friedrich-Alexander-Universität Erlangen-Nürnberg, Nikolaus-Fiebiger-Str. 10, 91058 Erlangen, Germany

*To whom correspondence should be addressed: dorothee.weikert@fau.de

<u>This file contains:</u>

Supplementary Figures S1 – S6

Supplementary Tables S1 – S5

Supplementary Methods

Supplementary References

Supplementary Figures

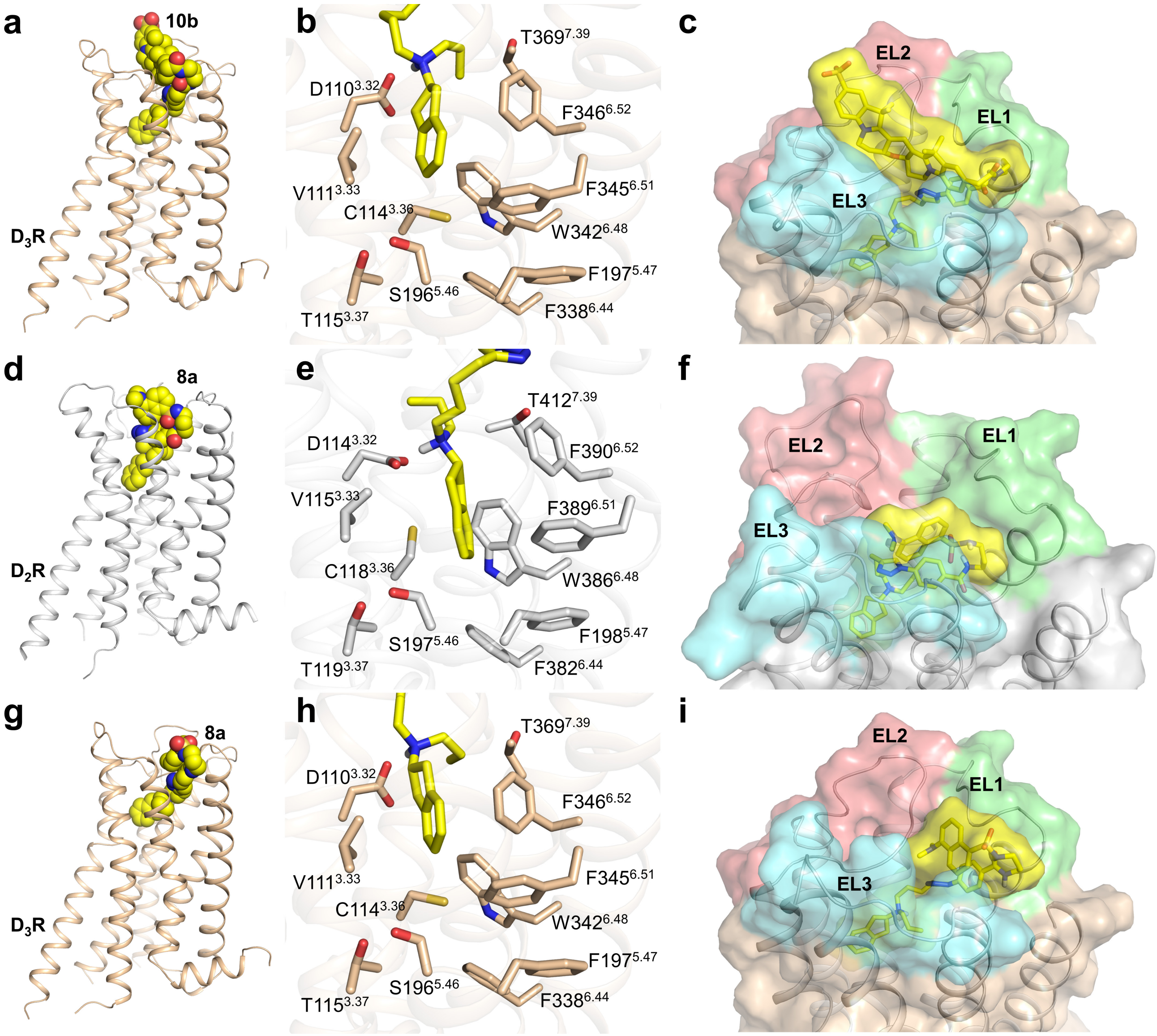

Supplementary Fig. 1. Docking poses of fluorescent ligands 8a and 10b. (**a-c**) The docking pose of **10b** in D_3R reveals highly similar interactions as compared to the pose in the D_2R (see Fig. 2 for comparison), except for the *N*-propyl substituent, which shows a slightly different orientation. Docking of the dansyl-labeled ligand **8a** to D_2R (**d-f**) or D_3R (**g-i**) leads to very similar receptor ligand interactions as compared to the poses of **10b**. The smaller fluorophore is fully accommodated between the extracellular loops 1-3 in different orientations in D_2R and D_3R, but forms mainly hydrophobic interactions in both cases. Ligands are colored yellow, while EL1, EL2 and EL3 are colored in green, red and cyan, respectively.

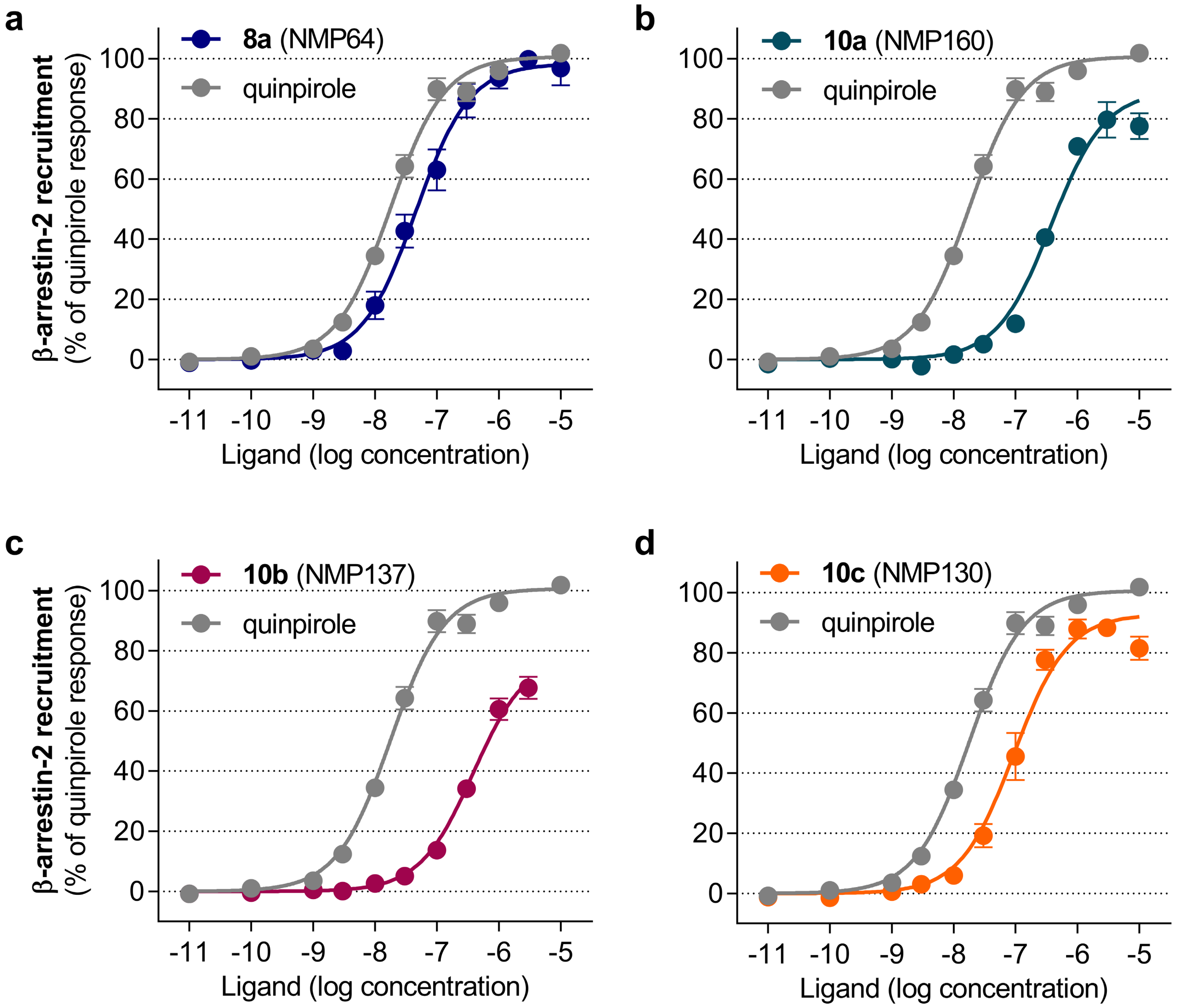

Supplementary Figure 2. β-arrestin-2 recruitment at $D_{2S}R$. Activation of $D_{2S}R$ upon stimulation with the fluorescent ligands **8a** (**a**) and **10a-c** (**b-d**) was determined employing the DiscoverX Pathhunter assay in HEK293 cells. The dansyl-labeled ligand **8a** possesses full agonist efficacy and only slightly reduced potency relative to the reference agonist quinpirole. Ligands **10a-c** comprising the larger trifluroethyl-rhodamine, Cy3B or Alexa488 fluorophores show slightly reduced potency and submaximal efficacy for the activation of $D_{2S}R$. Data represent mean ± S.E.M. of 5 – 10 individual experiments, each performed in duplicate.

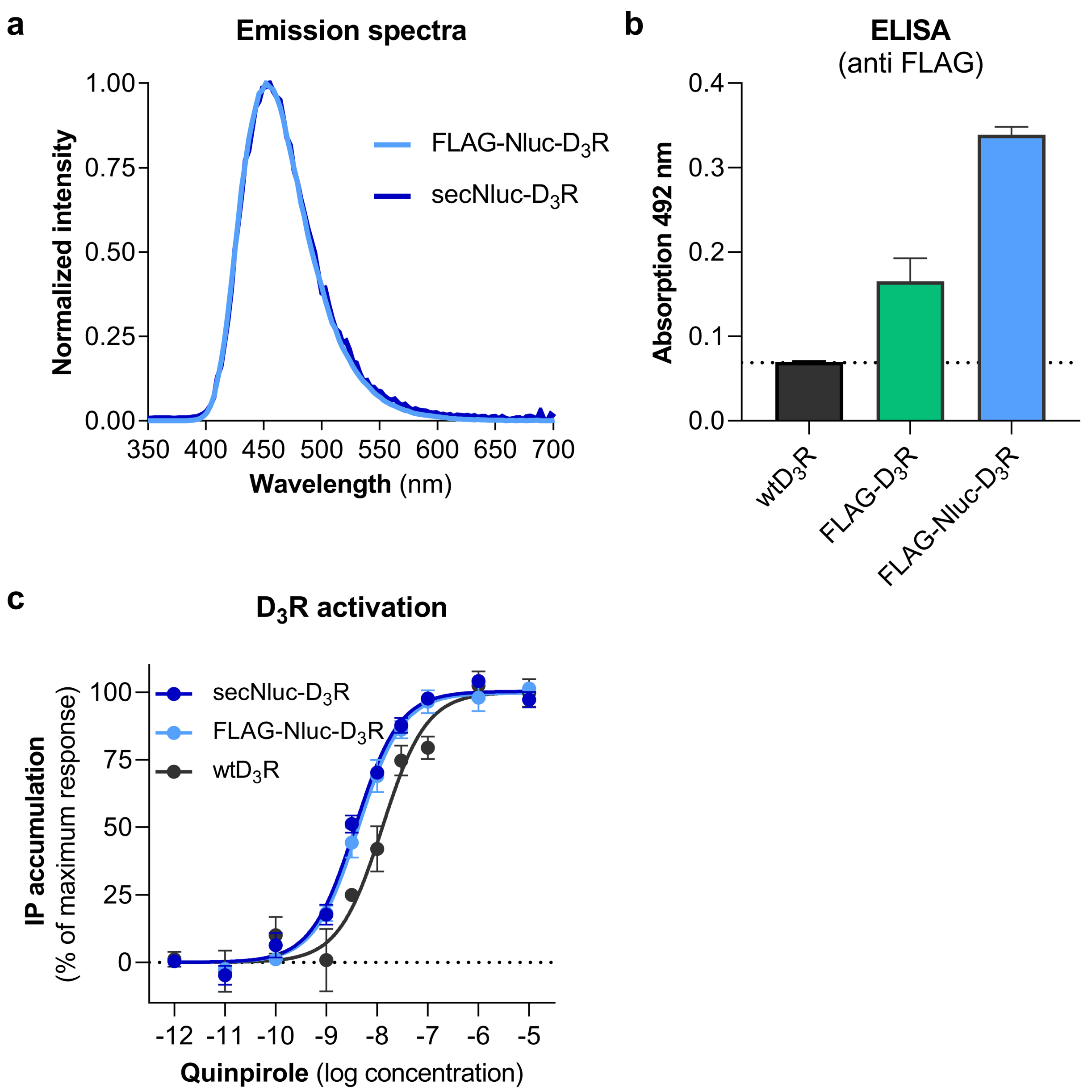

Supplementary Fig. 3. Characterization of Nluc-D$_3$R fusion proteins. (**a**) Comparison of the bioluminescence emission spectra of FLAG-Nluc-D$_3$R and secNluc-D$_3$R expressed in HEK293T cells obtained after addition of the luciferase substrate furimazine. Data show the normalized intensities of an individual experiment. (**b**) Surface expression of D$_3$R receptors is assessed by ELISA with a primary antibody directed against the N-terminal FLAG-tag and a secondary antibody conjugated to horse radish peroxidase. Due to the absence of the FLAG-tag, wtD$_3$R is not detected and serves as a negative control, indicating the baseline signal (dashed line). Data show mean ± SEM of three independent experiments, each performed in quadruplicate. (**c**) Concentration-response curves obtained for the reference agonist quinpirole in a second messenger-based activation assay with a promiscuous Gα_q protein[1] (IPOne, Cisbio) indicate that presence of the Nluc enzyme does not impair receptor activation. Data show mean ± SEM of four independent experiments, each performed in triplicate.

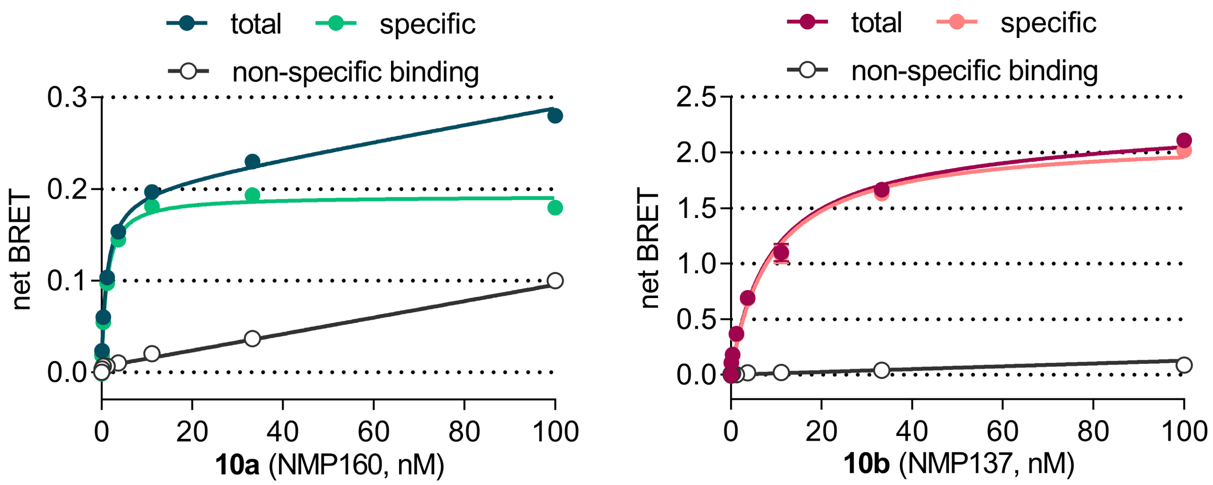

Supplementary Fig. 4. NanoBRET saturation curves obtained with ligands 10a,b and membranes from HEK293T expressing secNluc-D$_3$R. Experiments were carried out by incubating secNluc-D$_3$R membranes and serial dilutions (0-100 nM) of fluorescent ligands in the absence (total binding) or in the presence of 10 μM haloperidol (non-specific binding). Specific binding was calculated as the difference between total and non-specific binding. BRET was measured after 90 min incubation at 37 °C and represents mean ± SD of an individual representative out of 3 – 4 independent experiments, with each condition in triplicate.

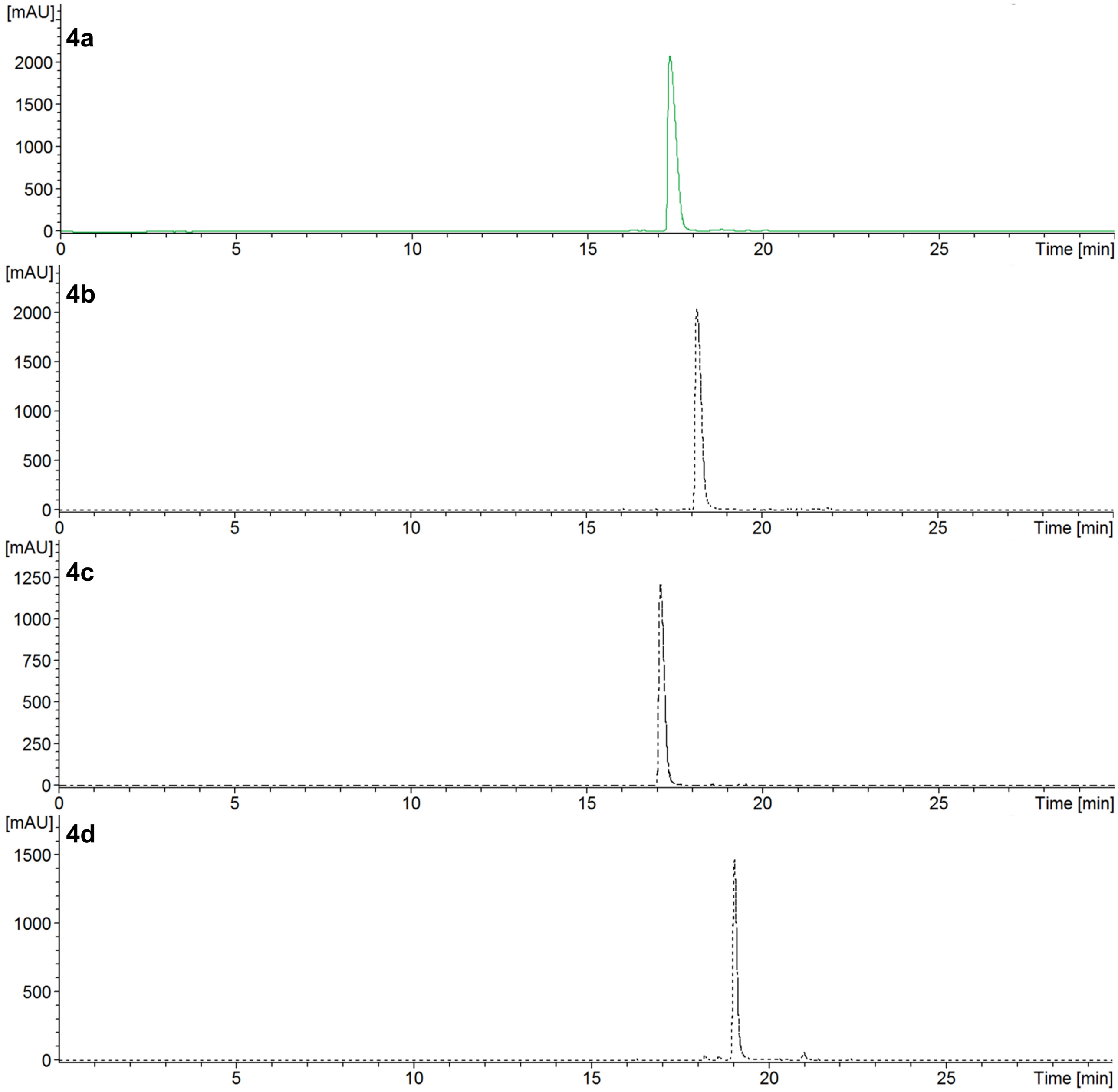

Supplementary Fig. 5. HPLC-Chromatograms of the fluorescent ligands 4a-d. Purity of the compounds was assessed by RP-HPLC with UV detection at 254 nm.

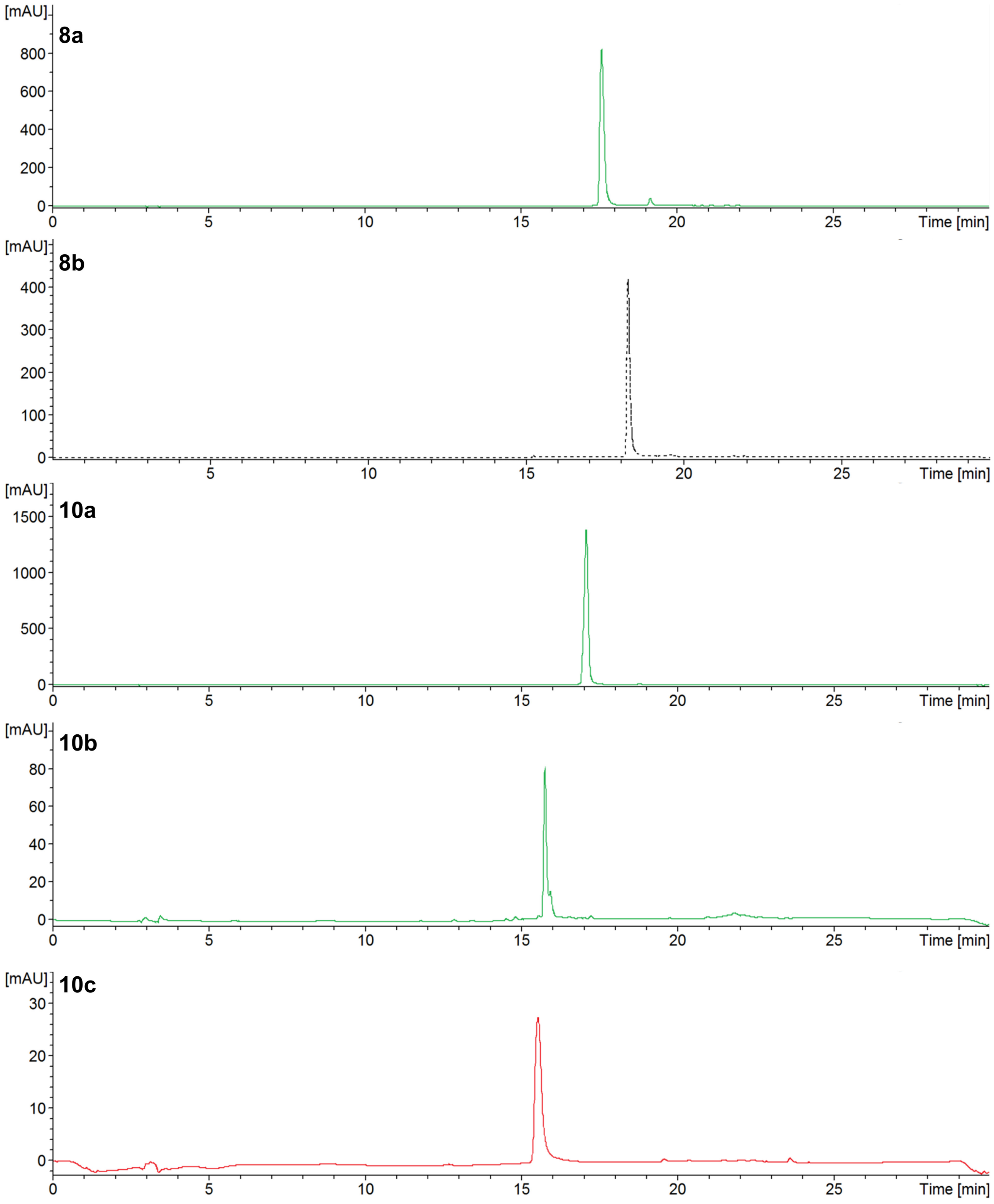

Supplementary Fig. 6. Chromatograms of the fluorescent ligands 8a,b and 10a-c. Purity of the compounds was assessed by RP-HPLC with UV detection at 254 nm.

Supplementary Tables

Supplementary Table S1. Binding affinities of the test compounds. Binding affinities of the test compounds for porcine D_1R, 5-$HT_{1A}R$, 5-HT_2R and α_1-AR were determined by radioligand competition.

	K_i [nM][a]			
	[^{3}H]SCH23,390	[^{3}H]WAY100,635	[^{3}H]ketanserin	[^{3}H]prazosin
	pD_1R	p5-HT_{1A}R	p5-HT_2R	pα_1-AR
**2a**	3,000 *(2)*<br>(1,400 – 4,600)	52 *(2)*<br>(33 – 70)	850 *(2)*<br>(710 – 990)	87 *(2)*<br>(44 - 130)
**2b**	540 *(2)*<br>(270 – 800)	260 *(2)*<br>(240 – 270)	1,100 *(3)*<br>(500 – 2,100)	4.9 *(2)*<br>(3.5 – 6.2)
**2c**	1,100 *(2)*<br>(1,000 -1,100)	25 *(2)*<br>(22 – 27)	600 *(2)*<br>(580 – 620)	2.4 *(2)*<br>(2.0 – 2.8)
**2d**	5,500 *(4)*<br>(3,700 – 8,400)	4,100 *(2)*<br>(2,600 – 5,600)	4,400 *(2)*<br>(3,900 – 4,900)	1,100 *(4)*<br>(630 – 1,900)
**4a**	590 *(2)*<br>(580 – 590)	27 *(2)*<br>(25 – 28)	220 *(2)*<br>(220 – 220)	29 *(2)*<br>(25 – 32)
**4b**	190 *(2)*<br>(140 – 240)	78 *(2)*<br>(46 – 110)	390 *(2)*<br>(42 – 730)	7.9 *(2)*<br>(6.1 – 9.7)
**4c**	180 *(2)*<br>(150 – 200)	55 *(2)*<br>(43 – 67)	450 *(2)*<br>(410 – 490)	1.1 *(2)*<br>(0.87 – 1.3)
**4d**	> 10,000 *(4)*<br>(15,000 – n.d.)	> 10,000 *(2)*<br>(n.d.)	> 10,000 *(2)*<br>(3,700 – n.d.)	3,000 *(4)*<br>(1,900 – 3,500)
**7a**	3,600 *(6)*<br>(2,300 – 4,900)	13 *(4)*<br>(9.9 – 17)	400 *(4)*<br>(320 – 520)	37 *(3)*<br>(34 – 38)
**8a**<br>(NMP64)	1,100 *(4)*<br>(520 – 1,700)	70 *(2)*<br>(50 – 89)	690 *(2)*<br>(550 – 830)	22 *(4)*<br>(17 – 26)
**8b**	340 *(2)*<br>(280 – 400)	170 *(2)*<br>(160 – 170)	380 *(2)*<br>(310 – 440)	23 *(2)*<br>(22 – 24)
**10a**<br>(NMP160)	310[b] *(2)*<br>(280 – 330)	n.t.[c]	n.t.[c]	100 *(2)*<br>(80 – 120)
**10b**<br>(NMP137)	5,100 *(4)*<br>(2,100 – 8,200)	120 *(2)*<br>(64 – 180)	330 *(2)*<br>(250 – 410)	87 *(4)*<br>(74 – 110)
**10c**<br>(NMP130)	5,100 *(3)*<br>(4,800 – 5,400)	85 *(2)*<br>(72 – 98)	2,200 *(2)*<br>(1,700 – 2,600)	150 *(2)*<br>(120 – 170)

[a] Data are indicated as mean (and range) of (*n*) individual experiments, each performed in triplicate; [b] assay performed with human isoform of D_1R; [c] n. d. K_i not determined as no complete ligand displacement occurred; [d] n.t. not tested.

Supplementary Table S2. Comparison of binding affinities for haloperidol, fluspirilene, aripiprazole and cariprazine for wtD_3R, FLAG-Nluc-D_3R and secNluc-D_3R. Binding affinities were determined using membranes from HEK293T cells expressing the individual receptor construct and the radioligand [^{3}H]spiperone.

	K_i (nM)[a]		
	wtD_3R	FLAG-Nluc-D_3R	secNluc-D_3R
haloperidol	4.2 ± 3.1	12.5 ± 8.0	5.5 ± 2.6
fluspirilene	0.12 ± 0.02	0.22 ± 0.03	0.24 ± 0.06
cariprazine	0.16 ± 0.02	0.48 ± 0.08	0.31 ± 0.08
aripiprazole	1.8 ± 0.3	3.7 ± 0.7	3.7 ± 1.0
n	*4*	*3*	*10*

[a] Data indicated as mean ± SEM deduced from *n* independent experiments, each performed in triplicates.

Supplementary Table S3. Kinetic parameters determined in association and dissociation assays with secNluc-D_3R and fluorescent ligand 10a performed at room temperature.

	K_{on} ($min^{-1} \times M^{-1}$)[a]	K_{off} (min^{-1})[a]	residence time (min)[a]
**10a**	(1.02 ± 0.30) × 10^7	0.054 ± 0.002	19 ± 1
n	*10*	*3*	*3*

[a] Data indicated as mean ± SEM deduced from *n* independent experiments, each performed in duplicates.

Supplementary Table S4. Binding affinities for haloperidol, fluspirilene, aripiprazole and cariprazine determined by NanoBRET between sec-Nluc-D_3R and the fluorescent ligand 10a.

	K_i (nM)[a]	
compound	**10a** (10 nM)	**10a** (100 nM)
haloperidol	14.8 ± 4.2	12.8 ± 3.5
fluspirilene	0.41 ± 0.12	0.37 ± 0.05
cariprazine	0.21 ± 0.07	0.21 ± 0.03
aripiprazole	11.5 ± 3.9	11.2 ± 2.5

[a] Data indicated as mean ± SEM deduced from 4-5 independent experiments, each performed in triplicates.

Supplementary Table S5. Experimental conditions for radioligand competition experiments.

Receptor	Radioligand (specific activity)	Radioligand concentration (nM)	K_D (nM)	B_{max} (fmol · mg^{-1} protein)	Protein concentration (μg/well)
pD_1R	[^{3}H]SCH23390 (80 Ci · $mmol^{-1}$) Biotrend	0.38 – 0.52	0.56 – 0.67	415 – 625	20 – 30
$hD_{2S}R$	[^{3}H]spiperone (73 Ci · $mmol^{-1}$) Perkin Elmer	0.09 – 0.23	0.040 – 0.11	2,600 – 5,000	1 – 4
$hD_{2L}R$		0.18 – 0.23	0.051 – 0.12	290 – 2,000	3 – 20
hD_3R		0.20 – 0.41	0.12 – 0.41	2,250 – 7,900	1 – 4
hD_4R		0.20 – 0.33	0.14 – 0.26	630 – 2,640	2 – 10
$p\alpha_1$-AR	[^{3}H]prazosin (84 Ci · $mmol^{-1}$) Perkin Elmer	0.18 – 0.23	0.070 – 0.14	73 – 235	30 – 60
p5-$HT_{1A}R$	[^{3}H]WAY100,635 (80 Ci · $mmol^{-1}$) Biotrend	0.16 – 0.28	0.048 – 0.15	50 – 90	60 – 100
p5-HT_2R	[^{3}H]ketanserin (47 Ci · $mmol^{-1}$) Perkin Elmer	0.46 – 0.88	1.75 – 2.20	150 – 530	80

Supplementary Methods

Organic Synthesis

Cy3B *N*-hydroxysuccinimide (NHS) ester was purchased from Amersham Biosciences. Alexa488 tetrafluorophenyl (TFP) ester was purchased from Molecular Probes. Other reagents were obtained from Sigma-Aldrich and Acros Organics. All reactions requiring anhydrous conditions were carried out under nitrogen atmosphere and the solvents were dried appropriately before use. Reactions were monitored by TLC using Merck Silica 60 F254 aluminum plates using UV fluorescence (254 nm) or $KMnO_4$ staining for detection. Column chromatography was carried out over Silica 60 and yields are reported after purification. ^{1}H, ^{13}C NMR spectra were recorded on a Bruker Avance 360 or a Bruker Avance 600 spectrometer at 300 K in the deuterated solvents indicated. Chemical shifts (δ) are given in parts per million (ppm) relative to TMS. Coupling constants (*J*) are measured in Hertz (Hz). The following abbreviations are used to describe multiplicities: s = singlet, d = doublet, t = triplet, q = quartet, b = broad, m = multiplet. Purity and identity were assessed by analytical RP-HPLC (Agilent 1100 analytical series, column: Zorbax Eclipse XDB-C8 (4.6 × 150 mm, 5 µm)) with the following binary solvent system: eluent methanol/0.1% aqueous formic acid, 10% methanol for 3 min, to 100% in 15 min, 100% for 6 min, to 10% in 3 min, then 10% for 3 min flow rate: 0.5 mL/min, detection wavelength: 254 nm coupled to a Bruker Esquire 2000 mass detector equipped with an ESI- or APCI-trap. HRMS was conducted at the Chair of Organic Chemistry, FAU Erlangen-Nürnberg on a Bruker maXis or micrOTOF instrument with APPI or ESI as the ionization method.

***N*-(Hex-5-yn-1-yl)-*N*-propyl-2,3-dihydro-1*H*-inden-2-amine (1a).** Following GP I, from *N*-propyl-2,3-dihydro-1*H*-inden-2-amine hydrochloride (0.5 g, 2.36 mmol) and 6-chlorohex-1-yne (0.43 mL, 3.54 mmol), title compound **1a** was prepared and isolated as black liquid (409 mg, 68%) after flash column chromatography purification (EtOAc/Cyclohexane, 1:9). ^{1}H-NMR (360 MHz, $CDCl_3$): δ 0.88 (t, *J* = 7.2 Hz, 3H), 1.45-1.62 (m, 6H), 1.93 (t, *J* = 2.8 Hz, 1H), 2.19-2.24 (m, 2H), 2.48-2.57 (m, 4H), 2.85-2.92 (m, 2H), 2.98-3.05 (m, 2H), 3.61-3.71 (m, 1H), 7.10-7.17 (m, 4H). ^{13}C-NMR (90 MHz, $CDCl_3$): δ 11.9, 18.3, 20.2, 26.1, 26.4, 36.5, 50.7, 53.2, 63.0, 68.3, 84.3, 124.3, 126.2, 141.8.

1-(Hex-5-yn-1-yl)-4-(2,3-dichlorophenyl)piperazine (1b). Following GP I, from 2,3-dichlorophenylpiperazine hydrochloride (1.0 g, 3.73 mmol) and 6-chlorohex-1-yne (0.54 mL,

4.5 mmol), title compound **1b** was prepared and isolated as yellow solid (889 mg, 76%) after flash column chromatography purification (EtOAc/Cyclohexane, 1:9). ^{1}H-NMR (600 MHz, $CDCl_3$): δ 1.55-1.60 (m, 2H), 1.61-1.67 (m, 2H), 1.94 (t, J = 2.4 Hz, 1H), 2.23 (td, J = 3.0 Hz, J = 7.2 Hz, 2H), 2.41-2.44 (m, 2H), 2.63 (bs, 4H), 3.06 (bs, 4H), 6.94-6.96 (m, 1H), 7.11-7.15 (m, 2H). ^{13}C-NMR (150 MHz, $CDCl_3$): δ 18.3, 25.9, 26.4, 51.3, 53.3, 58.0, 68.4, 84.3, 118.5, 124.5, 127.4, 127.5, 134.0, 151.3.

1-(Hex-5-yn-1-yl)-4-(2-methoxyphenyl)piperazine (1c). Following GP I, from 2-methoxyphenylpiperazine (0.5 g, 2.6 mmol) and 6-chlorohex-1-yne (0.4 mL, 3.9 mmol) title compound **1c** was prepared and isolated as gummy yellow solid (549 mg, 74%) after flash column chromatography purification (EtOAc/Cyclohexane, 1:9). ^{1}H-NMR (600 MHz, $CDCl_3$): δ 1.54-1.59 (m, 2H), 1.62-1.67 (m, 2H), 1.95 (t, J = 3.0 Hz, 1H), 2.22 (td, J = 3.0 Hz, J = 7.2 Hz, 2H), 2.41 (t, J = 7.8 Hz, 2H), 2.62 (bs, 4H), 3.09 (bs, 4H), 3.84 (s, 3H), 6.83-6.85 (m, 1H), 6.89-6.99 (m, 3H). ^{13}C-NMR (150 MHz, $CDCl_3$): δ 18.1, 25.7, 26.3, 50.4, 53.2, 55.1, 57.9, 68.3, 84.1, 111.0, 117.9, 120.8, 122.6, 141.2, 152.0.

2-(*tert*-Butyl)-4-(4-(hex-5-yn-1-yl)piperazin-1-yl)-6-(trifluoromethyl)pyrimidine (1d). Following GP I, from 2-(tert-butyl)-4-(piperazin-1-yl)-6-(trifluoromethyl)pyrimidine (1.0 g, 3.46 mmol) and 6-chlorohex-1-yne (0.4 mL, 3.46 mmol), title compound **1d** was prepared and isolated as liquid (1.08g, 85%) after flash column chromatography purification (EtOAc/Cyclohexane, 1:9). ^{1}H-NMR (360 MHz, $CDCl_3$): δ 1.34 (s, 9H), 1.54-1.72 (m, 4H), 1.96 (t, J = 2.5 Hz, 1H), 2.24 (td, J = 2.8 Hz, J = 6.8 Hz, 2H), 2.44 (t, J = 6.8 Hz, 2H), 2.55 (bs, 4H), 3.74 (bs, 4H), 6.58 (s, 1H). ^{13}C-NMR (90 MHz, $CDCl_3$): δ 18.5, 25.8, 26.4, 29.5, 39.6, 43.9, 52.8, 58.0, 68.8, 84.3, 95.7, 121.5, 154.2, 162.3, 177.9.

4-(4-(4-((2,3-Dihydro-1*H*-inden-2-yl)(propyl)amino)butyl)-1*H*-1,2,3-triazol-1-yl)benzonitrile (2a). Following GP II, from 4-azidobenzonitrile (170 mg, 1.17 mmol) and **1a** (300 mg, 1.17 mmol), title compound **2a** (433 mg, 92%) was prepared. ^{1}H-NMR (600 MHz, $CDCl_3$): δ 0.87 (t, J = 7.2 Hz, 3H), 1.46-1.52 (m, 2H), 1.56-1.60 (m, 2H), 1.73-1.78 (m, 2H), 2.48-2.50 (m, 2H), 2.58 (t, J = 7.8 Hz, 3H), 2.82-2.89 (m, 4H), 2.99-3.02 (m, 2H), 3.64-3.67 (m, 1H), 7.11-7.13 (m, 2H), 7.15-7.17 (m, 2H), 7.78 (s, 1H), 7.80-7.83 (m, 2H), 7.88-7.90 (m, 2H). ^{13}C-NMR (150 MHz, $CDCl_3$): δ 11.9, 20.2, 25.5, 29.9, 27.2, 36.5, 51.0, 53.4, 63.0, 112.0, 117.7, 118.4, 120.3, 124.4, 126.2, 133.8, 139.9, 141.8, 149.7; Purity: 99% (t_r: 15.6 min), LC-MS-APCI (m/z): $[M+H]^+$ calcd. for $C_{25}H_{29}N_5$, 400.5; found, 400.5.

4-(4-(4-(4-(2,3-Dichlorophenyl)piperazin-1-yl)butyl)-1*H*-1,2,3-triazol-1-yl)benzonitrile (2b). Following GP II, from 4-azidobenzonitrile (207 mg, 1.44 mmol) and **1b** (300 mg, 0.96 mmol), title compound **2b** (362 mg, 82%) was prepared. ^{1}H-NMR (600 MHz, $CDCl_3$): δ 1.63-1.67 (m, 2H), 1.76-1.82 (m, 2H), 2.48 (t, *J* = 7.2 Hz, 2H), 2.64 (bs, 4H), 2.86 (t, *J* = 7.8 Hz, 2H), 3.07 (bs, 4H), 6.89-6.97 (m, 1H), 7.12-7.18 (m, 2H), 7.79 (s, 1H), 7.79-7.91 (m, 4H). ^{13}C-NMR (150 MHz, $CDCl_3$): δ 25.4, 26.4, 27.1, 51.2, 53.3, 58.2, 112.0, 117.7, 118.4, 118.5, 120.3, 124.5, 127.4, 127.5, 133.8, 134.0, 139.9, 149.5, 151.2; Purity: 98% (t_r: 16.2 min), LC-MS-APCI (m/z): $[M+H]^+$ calcd. for $C_{23}H_{24}Cl_2N_6$, 456.3; found, 456.1.

4-(4-(4-(4-(2-Methoxyphenyl)piperazin-1-yl)butyl)-1*H*-1,2,3-triazol-1-yl)benzonitrile (2c). Following GP II, from 4-azidobenzonitrile (238 mg, 1.65 mmol) and **1c** (300 mg, 1.10 mmol), title compound **2c** (398 mg, 87%) was prepared. ^{1}H-NMR (600 MHz, $CDCl_3$): δ 1.59-1.64 (m, 2H), 1.74-1.79 (m, 2H), 2.43 (t, *J* = 7.2 Hz, 2H), 2.62 (bs, 4H), 2.82 (t, *J* = 7.2 Hz, 2H), 3.06, (bs, 4H), 3.82 (s, 3H), 6.82-6.97 (m, 4H), 7.78-7.87 (m, 5H). ^{13}C-NMR (150 MHz, $CDCl_3$): δ 25.7, 26.6, 27.3, 50.8, 53.6, 55.5, 58.2, 111.3, 112.1, 117.9, 118.3, 118.6, 120.4, 121.1, 123.0, 134.0, 140.1, 141.4, 149.7, 152.4; Purity: 98% (t_r: 15.0 min). LC-MS-APCI (m/z): $[M+H]^+$ calcd. for $C_{24}H_{28}N_6O$, 417.5; found, 417.5.

4-(4-(4-(4-(2-(tert-Butyl)-6-(trifluoromethyl)pyrimidin-4-yl)piperazin-1-yl)butyl)-1*H*-1,2,3-triazol-1-yl)benzonitrile (2d). Following GP II, from 4-azidobenzonitrile (37 mg, 0.27 mmol) and **1d** (100 mg, 0.27 mmol), title compound **2d** (118 mg, 86%) was prepared. ^{1}H-NMR (600 MHz, $CDCl_3$): δ 1.33 (s, 9H), 1.62-1.65 (m, 2H), 1.79-1.85 (m, 2H), 2.44 (t, *J* = 7.8 Hz, 2H), 2.50-2.53 (m, 4H), 2.86 (t, *J* = 7.8 Hz, 2H), 3.71 (bs, 4H), 6.58 (s, 1H), 7.80 (s, 1H), 7.82-7.91 (m, 4H). ^{13}C-NMR (150 MHz, $CDCl_3$): δ 25.4, 25.5, 26.3, 27.1, 29.3, 39.4, 43.8, 52.7, 58.1, 95.5, 112.1, 118.5, 120.3, 122.8, 133.8, 139.9, 149.5, 154.2, 162.1, 177.7; Purity: 99% (t_r: 18.5 min), LC-MS-APCI (m/z): $[M+H]^+$ *c*alcd. for $C_{26}H_{31}F_3N_8$, 513.2; found, 513.3

***N*-(4-(1-(4-(Aminomethyl)phenyl)-1*H*-1,2,3-triazol-4-yl)butyl)-*N*-propyl-2,3-dihydro-1*H*-inden-2-amine (3a); 4-(4-(4-(4-(2,3-Dichlorophenyl)piperazin-1-yl)butyl)-1*H*-1,2,3-triazol-1-yl)benzylamine (3b); 4-(4-(4-(4-(2-Methoxyphenyl)piperazin-1-yl)butyl)-1*H*-1,2,3-triazol-1-yl)benzylamine (3c); 4-(4-(4-(4-(2-(tert-Butyl)-6-(trifluoromethyl)pyrimidin-4-yl)piperazin-1-yl)butyl)-1*H*-1,2,3-triazol-1-yl)benzylamine (3d).** To a solution of the 1,3-cycloaddition products **2a-d** in anhydrous THF at 0 °C were added 2 equivalents of $LiAlH_4$ (4 M-THF) and the reaction mixture was stirred for several hours. Upon completion, the reaction was quenched by the dropwise addition of saturated $NaHCO_3$ at 0 °C, filtered over Na_2SO_4 and evaporated. The crude primary amines **3a-d** were used for the next step without further purification.

5-(Dimethylamino)-*N*-(4-(4-(4-((2,3-dihydro-1*H*-inden-2-yl)(propyl)amino)butyl)-1*H*-1,2,3-triazol-1-yl)benzyl)naphthalene-1-sulfonamide (4a). Following GP III, from **3a** (412 mg, 1.02 mmol) and dansyl chloride (827 mg, 3.07 mmol), title compound **4a** was isolated as green gummy solid (572 mg, 88%). ^{1}H-NMR (600 MHz, $CDCl_3$): δ 0.87 (t, *J* = 7.3 Hz, 3H), 1.48-1.55 (m, 2H), 1.62-1.64 (m, 2H), 1.71-1.76 (m, 2H), 2.55 (bs, 2H), 2.63 (bs, 2H), 2.78-2.81 (m, 2H), 2.85 (s, 6H), 2.91-2.96 (m, 2H), 3.01-3.05 (m, 2H), 3.66-3.72 (m, 1H),4.12 (d, *J* = 5.0 Hz, 2H), 5.39 (t, *J* = 5.0 Hz, 1H), 7.11-7.18 (m, 7H), 7.44-7.49 (m, 3H), 7.52-7.55 (m, 1H), 7.66 (s, 1H), 8.23-8.30 (m, 2H), 8.50-8.52 (m, 1H). ^{13}C-NMR (150 MHz, $CDCl_3$): δ 11.8, 19.7, 25.4, 27.2, 36.3, 45.3, 46.5, 51.0, 53.2, 53.3, 63.1, 115.2, 118.5, 118.7, 120.0, 123.1, 124.4, 126.3, 128.5, 128.9, 129.5, 129.8, 129.9, 130.6, 134.5, 136.4, 136.7, 141.5, 148.7, 152.0. Purity: 97% (t_r: 17.5 min). LC-MS-ESI (m/z): $[M+H]^+$ calcd. for $C_{37}H_{44}N_6O_2S$, 637.8; found, 637.4.

5-(Dimethylamino)-*N*-(4-(4-(4-(4-(2,3-dichlorophenyl)piperazin-1-yl)butyl)-1*H*-1,2,3-triazol-1-yl)benzyl)naphthalene-1-sulfonamide (4b). Following GP III, from **3b** (200 mg, 0.43 mmol) and dansyl chloride (350 mg, 1.29 mmol), title compound **4b** was isolated as green semi-solid (157 mg, 53%). ^{1}H-NMR (360 MHz, $CDCl_3$): δ 1.62-1.68 (m, 2H), 1.74-1.82 (m, 2H), 2.48 (t, *J* = 7.5 Hz, 2H), 2.65 (bs, 4H), 2.82 (t, *J* = 7.2 Hz, 2H), 2.87 (s, 6H), 3.07 (bs, 4H), 4.14 (d, *J* = 6.4 Hz, 2H), 5.03 (t, *J* = 6.4 Hz, 1H), 6.93-6.96 (m, 1H), 7.11-7.23 (m, 5H), 7.49-7.59 (m, 4H), 7.66 (s, 1H), 8.25-8.29 (m, 2H), 8.52-8.55 (m, 1H). ^{13}C-NMR (90 MHz, $CDCl_3$): δ 25.5, 26.3, 27.3, 45.3, 46.6, 51.2, 53.3, 58.2, 115.2, 118.4, 118.5, 118.7, 120.2, 123.1, 124.5, 127.4, 127.5, 128.5, 129.0, 129.5, 129.8, 129.9,

130.7, 134.0, 134.4, 136.5, 136.7, 148.7, 151.2, 152.1. Purity: 99% (t_r: 18.1 min). LC-MS-APCI (m/z): $[M+H]^+$ calcd. for $C_{35}H_{39}Cl_2N_7O_2S$, 692.7; found, 692.6.

5-(Dimethylamino)-*N*-(4-(4-(4-(4-(2-methoxyphenyl)piperazin-1-yl)butyl)-1*H*-1,2,3-triazol-1-yl)benzyl)naphthalene-1-sulfonamide (4c). Following GP III, from **3c** (778 mg, 1.85 mmol) and dansyl chloride (748 mg, 2.77 mmol), title compound **4c** was isolated as green semi-solid (880 mg, 73%). ^{1}H-NMR (360 MHz, $CDCl_3$): δ 1.58-1.69 (m, 2H), 1.71-1.82 (m, 2H), 2.46 (t, *J* = 7.9 Hz, 2H), 2.65 (bs, 4H), 2.82 (t, *J* = 7.5 Hz, 2H), 2.87 (s, 6H), 3.09 (bs, 4H), 3.85 (s, 3H), 4.14 (d, *J* = 5.7 Hz, 2H), 5.16 (t, *J* = 5.9 Hz, 1H), 6.84-7.01 (m, 4H), 7.17-7.22 (m, 3H), 7.46-7.58 (m, 4H), 7.65 (s, 1H), 8.22-8.30 (m, 2H), 8.51-8.54 (m, 1H). ^{13}C-NMR (90 MHz, $CDCl_3$): δ 25.5, 25.6, 26.4, 27.3, 45.3, 46.6, 50.5, 53.5, 55.3, 58.4,111.2, 115.2, 118.4, 118.6, 120.1, 120.9, 122.8, 123.1, 128.5, 128.9, 129.5, 129.9, 130.7, 134.5, 136.5, 136.7, 141.3, 148.8, 152.1, 152.2. Purity: 99% (t_r: 17.1 min). LC-MS-APCI (m/z): $[M+H]^+$ *c*alcd. for $C_{36}H_{43}N_7O_3S$, 654.8; found, 654.8.

5-(Dimethylamino)-*N*-(4-(4-(4-(4-(2-(tert-butyl)-6-(trifluoromethyl)pyrimidin-4-yl)piperazin-1-yl)butyl)-1*H*-1,2,3-triazol-1-yl)benzyl)naphthalene-1-sulfonamide (4d). Following GP III, from **3d** (440 mg, 0.85 mmol) and dansyl chloride (276 mg, 1.02 mmol), title compound **4d** was isolated as green solid (498 mg, 78%). ^{1}H-NMR (600 MHz, $CDCl_3$): δ 1.33 (s, 9H), 1.59-1.64 (m, 2H), 1.75-1.80 (m, 2H), 2.42 (t, *J* = 7.2 Hz, 2H), 2.50 (t, *J* = 4.8 Hz, 4H), 2.81 (t, *J* = 7.8 Hz, 2H), 2.86 (s, 6H), 3.69 (bs, 4H), 4.12 (d, *J* = 6.6 Hz, 2H), 5.42-5.44 (m, 1H), 6.57 (s, 1H), 7.16-7.20 (m, 3H), 7.47-7.55 (m, 4H), 7.65 (s, 1H), 8.24-8.30 (m, 2H), 8.51 (d, *J* = 8.4 Hz, 1H). ^{13}C-NMR (150 MHz, $CDCl_3$): δ 25.6, 26.5, 27.4, 29.5, 39.6, 44.0, 45.5, 46.7, 52.9, 58.3, 95.7, 115.4, 118.7, 118.9, 120.3, 120.6, 122.4, 123.3, 128.7, 129.2, 129.7, 130.0, 130.1, 130.8, 134.7, 136.6, 137.1, 148.9, 152.2, 162.2, 177.8. Purity: 97% (t_r: 19.0 min). LC-MS-APCI (m/z): $[M+H]^+$ calcd. for $C_{38}H_{46}F_3N_9O_2S$, 750.8; found, 750.6.

4-(4-(4-((2,3-Dihydro-1*H*-inden-2-yl)(propyl)amino)butyl)-1*H*-1,2,3-triazol-1-yl)benzoic acid (5a). Following GP II, from 4-azidobenzoic acid (50 mg, 0.30 mmol) and **1a** (76 mg, 0.30 mmol), title compound **5a** was prepared (95 mg, 76%).

4-(4-(4-(4-(2,3-Dichlorophenyl)piperazin-1-yl)butyl)-1*H*-1,2,3-triazol-1-yl)benzoic acid (5b). Following GP II, from 4-azidobenzoic acid (50 mg, 0.30 mmol) and **1b** (95 mg, 0.30 mmol), title compound **5b** was prepared (118 mg, 83%).

***tert*-Butyl 3-(4-(4-(4-((2,3-dihydro-1*H*-inden-2-yl)(propyl)amino)butyl)-1*H*-1,2,3-triazol-1-yl)benzamido)propylcarbamate (6a).** Following GP IV, from **5a** (50 mg, 0.12 mmol), DIPEA (0.04 mL, 0.22 mmol), TBTU (42.0 mg, 0.13 mmol) and tert-butyl 3-aminopropylcarbamate (20 mg, 0.12 mmol), title compound **6a** was prepared (60 mg, 88%).

***tert*-Butyl 3-(4-(4-(4-(4-(2,3-dichlorophenyl)piperazin-1-yl)butyl)-1*H*-1,2,3-triazol-1-yl)benzamido)propylcarbamate (6b).** Following GP IV, from **5b** (100 mg, 0.22 mmol), DIPEA (0.08 mL, 0.44 mmol), TBTU (84.0 mg, 0.26 mmol) and tert-butyl 3-aminopropylcarbamate (38 mg, 0.22 mmol), title compound **6b** was prepared (117 mg, 85%).

3-(4-(4-(4-((2,3-Dihydro-1*H*-inden-2-yl)(propyl)amino)butyl)-1*H*-1,2,3-triazol-1-yl)benzamido)propan-1-ammonium chloride (7a). 6a (100 mg, 0.17 mmol) was treated with 4 M HCl dioxane (0.7 mL, 4 mL per 1 mmol) over night at room temperature. Removal of excess of HCl dioxane under vacuum yielded title compound **7a** (85 mg, 98%) as HCl salt. ^{1}H-NMR (600 MHz, DMSO-d6): δ 0.92 (t, *J* = 7.8 Hz, 3H), 1.70-1.91 (m, 6H), 2.8 (t, *J* = 7.2 Hz, 2H), 2.85-2.87 (m, 2H), 2.92 (bs, 2H), 3.03-3-08 (m, 2H), 3.14-3.18 (m, 2H), 3.24-3.39 (m, 6H), 4.16-4.23 (m, 1H), 7.19-7.25 (m, 4H), 8.01-8.03 (m, 2H), 8.07 (bs, 3H), 8.11-8.12 (m, 2H), 8.76 (s, 1H), 8.95 (t, *J* = 5.4 Hz, 1H), 11.07 (bs, 1H). ^{13}C-NMR (90 MHz, DMSO-d6): δ 11.0, 22.1, 24.3, 25.7, 27.2, 34.3, 36.3, 36.7, 49.8, 51.6, 54.9, 62.8, 119.2, 120.4, 124.3, 127.0, 129.0, 133.7, 138.5, 139.2, 147.8, 165.3 LC-MS-ESI (m/z): $[M+H]^+$ *c*alcd. for $C_{28}H_{38}N_6O$, 475.3; found, 475.3.

3-(4-(4-(4-(4-(2,3-Dichlorophenyl)piperazin-1-yl)butyl)-1*H*-1,2,3-triazol-1-yl)benzamido)propan-1-ammmonium chloride (7b). 6b (100 mg, 0.15 mmol) was treated with 4 M HCl dioxane (0.6 mL; 4 mL per 1 mmol) over night at room temperature. Removal of excess of HCl dioxane under vacuum yielded title compound **7b** (90 mg, 98%) as HCl salt. ^{1}H-NMR (600 MHz, $CDCl_3$): δ 1.62-1.67 (m, 2H), 1.76-1.82 (m, 4H), 2.47 (t, *J* = 7.8 Hz, 2H), 2.63 (bs, 4H), 2.85 (t, *J* = 7.8 Hz, 2H), 2.98 (t, *J* = 4.8 Hz, 2H), 3.06 (bs, 4H), 3.61-3.64 (m, 2H), 6.94-6.95 (m, 1H), 7.12-7.16 (m, 2H), 7.78-7.80 (m, 3H), 7.96-7.97 (m, 2H), 8.24 (bs, 1H). ^{13}C-NMR (90 MHz, $CDCl_3$): δ 25.5, 26.5, 27.3, 30.6, 40.1, 41.2, 51.3, 53.3, 58.2, 118.5, 118.6, 119.9, 124.5, 127.4, 127.5, 128.6, 134.0, 134.6, 139.0, 149.1, 151.3, 165.8. LC-MS-ESI (m/z): $[M+H]^+$ *c*alcd. for $C_{26}H_{33}Cl_2N_7O$, 530.2; found, 530.3.

4-(4-(4-((2,3-Dihydro-1*H*-inden-2-yl)(propyl)amino)butyl)-1*H*-1,2,3-triazol-1-yl)-*N*-(3-(5-(dimethylamino)naphthalene-1-sulfonamido)propyl)benzamide (8a). Following GP III, from **7a** (100 mg, 0.20 mmol) and dansyl chloride (80 mg, 0.30 mmol), title compound **8a** was isolated as green gummy solid (103 mg, 73%). ^{1}H-NMR (360 MHz, $CDCl_3$): δ 0.87 (t, J = 7.2 Hz, 3H), 1.48-1.57 (m, 2H), 1.65-1.78 (m, 6H), 2.56-2.60 (m, 2H), 2.68 (bs, 2H), 2.76-2.84 (m, 8H), 2.94-3.08 (m, 6H), 3.46-3.51 (m, 2H), 3.67-3.76 (m, 1H), 5.23 (t, J = 6.1 Hz, 1H), 7.09-7.15 (m, 5H), 7.20 (t, J = 6.1 Hz, 1H), 7.44-7.52 (m, 2H), 7.69 (d, J = 8.6 Hz, 2H), 7.81 (s, 1H), 7.87 (d, J = 9.0 Hz, 2H), 8.19 (dd, J = 1.0 Hz, J = 7.2 Hz, 1H), 8.34 (d, J = 8.6 Hz, 1H), 8.49 (d, J = 8.2 Hz, 1H). ^{13}C-NMR (90 MHz, $CDCl_3$): δ 11.7, 19.5, 25.3, 27.0, 29.3, 36.2, 36.4, 40.0, 45.3, 51.0, 53.1, 53.3, 63.1, 115.1, 118.8, 118.9, 119.7, 123.1, 124.3, 126.4, 128.3, 128.7, 129.0, 129.5, 129.8, 130.3, 133.9, 135.1, 139.0, 141.1, 148.9, 151.9, 166.5. Purity: 99% (t_r: 17.7 min). LC-MS-ESI (m/z): $[M+H]^+$ calcd. for $C_{40}H_{49}N_7O_3S$, 708.4; found, 708.4. HRMS-APPI (m/z): $[M+H]^+$ calcd. for $C_{40}H_{49}N_7O_3S$, 708.3690; found, 708.3691.

4-(4-(4-(4-(2,3-Dichlorophenyl)piperazin-1-yl)butyl)-1*H*-1,2,3-triazol-1-yl)-*N*-(3-(1-(dimethylamino)naphthalene-5-sulfonamido)propyl)benzamide (8b). Following GP III, from **7b** (90 mg, 0.17 mmol) and dansyl chloride (137 mg, 0.51 mmol), title compound **8b** was isolated as green semi-solid (93 mg, 72%). ^{1}H-NMR (600 MHz, $CDCl_3$): δ 1.64-1.66 (m, 2H), 1.72-1.74 (m, 2H), 1.79-1.82 (m, 2H), 2.48 (t, J = 7.8 Hz, 2H), 2.65 (bs, 4H), 2.85 (t, J = 7.8 Hz, 2H), 2.88 (s, 6H), 2.99-3.02 (m, 2H), 3.07 (bs, 4H), 3.56-3.59 (m, 2H), 5.51 (t, J = 6.6 Hz, 1H), 6.77 (t, J = 6.6 Hz, 1H), 6.94-6.95 (m, 1H), 7.12-7.19 (m, 3H), 7.49-7.51 (m, 1H), 7.56-7.59 (m, 1H), 7.79-7.81 (m, 3H), 7.90-7.92 (m, 2H), 8.21-8.22 (m, 1H), 8.31-8.32 (m, 2H), 8.53-8.55 (m, 1H). ^{13}C-NMR (90 MHz, $CDCl_3$): δ 25.5, 26.4, 27.2, 29.4, 36.4, 40.1, 45.3, 51.2, 53.3, 58.2, 115.2, 118.5, 118.6, 118.7, 119.9, 123.1, 124.5, 127.4, 127.5, 128.5, 128.6, 129.2, 129.5, 129.9, 130.5, 133.9, 134.0, 134.8, 139.2, 149.1, 151.2, 152.1, 166.4. Purity: 99% (t_r: 18.1 min). LC-MS-ESI (m/z): $[M+H]^+$ calcd. for $C_{38}H_{44}Cl_2N_8O_3S$, 763.2; found, 763.4.

5´-Carboxy-*N*,*N*´-bis(2,2,2-trifluoroethyl)rhodamine (9).[2] A mixture of benzene-1,2,4-tricarboxylic acid anhydride (1.27 g, 6.6 mmol) and 3-[(2,2,2-trifluorethyl)aminophenol (1.06 g, 5.5 mmol) was stirred at 170 °C for 3 h. After addition of another portion of 3-[(2,2,2-trifluorethyl)aminophenol (0.84 g, 4.4 mmol) and 5.0 mL H_3PO_4, the mixture was stirred for another 3 h at 170 °C. The reaction

mixture was cooled to room temperature, 10 mL MeOH and 2 mL H_2O were added, and the suspension was stirred over night. The product was isolated by reverse phase flash chromatography (RP-C18, MeOH/0.1 % aqueous formic acid, gradient 10-60 %; 0.5 % per CV) as orange-red solid, (853 mg, 24%). ^{1}H-NMR (600 MHz, DMSO-d6): δ 3.95-4.04 (m, 4H), 6.48 (d, *J* = 8.7 Hz, 2H), 6.50 (dd, *J* = 8.7 Hz, *J* = 1.9 Hz, 2H), 6.65 (d, *J* = 1.9 Hz, 2H), 6.76 (t, *J* = 7.0 Hz, 2H), 7.32 (d, *J* = 7.9 Hz, 1H), 8.28 (dd, *J* = 7.9, 1.5 Hz, 1H), 8.37 (s, 1H). Purity: 96% (t_r: 17.5 min). LC-MS-ESI (m/z): $[M+H]^+$ calcd. for $C_{25}H_{16}F_6N_2O_5$, 539.1; found, 539.2.

***N*-(4-(1-(4-(Aminomethyl)phenyl)-1*H*-1,2,3-triazol-4-yl)butyl)-*N*-propyl-2,3-dihydro-1*H*-inden-2-amine-5´-carboxy-*N*,*N*´-bis(2,2,2-trifluoroethyl)rhodamine conjugate (10a).** To a solution of 10 mg (19.0 µmol) 5´-carboxy-*N*,*N*´-bis(2,2,2-trifluoroethyl)rhodamine (**9**)[2] and DIPEA (38 µmol, 6 µL) in CH_2Cl_2 at 0 ºC was added TBTU (19.0 µmol, 6 mg) in anhydrous DMF and the mixture was stirred for 30 min. A solution of the primary amine **3a** in CH_2Cl_2 was added to the above mixture and stirred for 24 hours. Title compound **10a** was purified by preparative RP-HPLC with detection wavelength of 495 nm (eluent: 0.1% HCO_2H in CH_3CN (A) + H_2O (B) applying a linear gradient 20-95% A in 80-5% B in 16 min, t_r: 12.3 min) and subsequent lyophilization to afford the conjugate as the formate salt (3.0 mg, 23%). Purity and identity were assessed by analytical HPLC coupled to a Bruker Esquire 2000 mass detector equipped with an ESI-trap, purity: 99% (t_r: 17.1 min). LC-MS-ESI (m/z): $[M+H]^+$, $[M+2H]^{2+}$ calcd. for $C_{50}H_{47}F_6N_7O_4$, 924.3, 462.5; found, 924.4, 462.9. HRMS-APPI (m/z): $[M+H]^+$, $[M+Na]^+$ calcd. for $C_{50}H_{47}F_6N_7O_4$, 924.3666, 946.3486; found, 924.3674; 946.3484.

***N*-(3-Aminopropyl)-4-(4-(4-((2,3-dihydro-1*H*-inden-2-yl)(propyl)amino)butyl)-1*H*-1,2,3-triazol-1-yl)benzamide-Cy3B conjugate (10b).** Aliquots of a solution of the primary amine **7a** (1.0 mg, 2.1 µmol) in dry DMF (0.5 mL) and DIPEA (5.6 µmol, 1.0 µL) were added to a vial of Cy3B *N*-hydroxysuccinimide (NHS) ester (1 mg, 1.06 µmol). The vial was kept at room temperature for 24 h. Title compound **10b** was isolated using preparative RP-HPLC with a detection wavelength of 565 nm (eluent: 0.1% HCO_2H in CH_3CN (A) + H_2O (B) applying a linear gradient 20-95% A in 80-5% B in 16 min, t_r: 11.7 min) and subsequent lyophilization to afford the conjugate as the formate salt (0.75 mg, 66%). Purity and identity was assessed by analytical HPLC coupled to a Bruker Esquire 2000 mass detector equipped with an ESI-trap. Purity: 99% (t_r: 15.7 min), LC-MS-ESI (m/z): $[M+2H]^{2+}$

calcd. for $C_{59}H_{68}N_8O_6S$, 509.7; found, 509.5. HRMS-APPI (m/z): $[M+H]^+$ calcd. for $C_{59}H_{68}N_8O_6S$, 1017.5055; found, 1017.5059.

***N*-(3-Aminopropyl)-4-(4-(4-((2,3-dihydro-1*H*-inden-2-yl)(propyl)amino)butyl)-1*H*-1,2,3-triazol-1-yl)benzamide-Alexa488 conjugate (10c).** Aliquots of a solution of the primary amine **7a** (0.6 mg, 1.2 µmol) in dry DMF (0.5 mL) and DIPEA (11.6 µmol, 1.8 µL) were added to a vial of Alexa488 TFP ester (1 mg, 1.13 µmol). The vial was kept at room temperature for 24 h. The reaction was carried out in triplicate. Title compound **10c** was isolated by preparative RP-HPLC with a detection wavelength of 495 nm (eluent: 0.1% HCO_2H in CH_3CN (A) + H_2O (B) applying a linear gradient 20-95% A in 80-5% B in 16 min, t_r: 10.6 min) and subsequent lyophilization to afford the conjugate as the formate salt (2.25 mg, 58%). Purity and identity was assessed by analytical HPLC coupled to a Bruker Esquire 2000 mass detector equipped with an ESI-trap, purity: 99% (t_r: 15.5 min). LC-MS-ESI (m/z): $[M+2H]^{2+}$ *c*alcd. for $C_{49}H_{50}N_8O_{11}S_2$, 496.5; found, 496.3. HRMS-ESI (m/z): $[M+H]^+$ *c*alcd. for $C_{49}H_{50}N_8O_{11}S_2$, 991.3113; found, 991.3117.

β-arrestin-2 recruitment

Recruitment of β-arrestin-2 to $D_{2S}R$ was determined employing the DiscoverX PathHunter assay (Eurofins, Freemont, USA) as described previously.[3] In brief, HEK293 cells stably expressing the enzyme acceptor (EA) tagged β-arrestin-2 fusion protein were transfected with the $D_{2S}R$-ARMS2-PK2 construct and human GRK2 using Mirus TransIT-293. 24 h after transfection, cells were detached using with Versene (Life Technologies), seeded into 384-well plates (white F-Bottom, Greiner BioOne, 5,000 cells/well) and maintained for 24 h at 37 °C, 5% CO_2. Cells were incubated with the test compounds diluted in PBS for 5 h at 37 °C, before the detection mix was added and incubation was continued for 60 min at room temperature. Chemiluminescence was determined using a CLARIOstar microplate reader (BMG Labtech, Ortenberg, Germany). Concentration-response curves were analyzed using the algorithms for three parameter non-linear regression (PRISM8.0) with all responses normalized to the maximum effect of quinpirole (100%) and vehicle (0%).

ELISA

Surface expression of FLAG-tagged D_3R was quantified in analogy to previously described protocols.[5] In brief, HEK293T cells were transfected with the plasmids encoding wtD_3R, FLAG-D_3R and FLAG-Nluc-D_3R using polyethyleneimine in suspension. Hence, HEK293T cells were detached from their culture plates, diluted to a density of 3 x 10^5 cells · mL^{-1} in DMEM/F12 supplemented with 10% FBS and mixed with the preformed transfection complex (PEI/DNA ratio 3:1) consisting of 0.5 µg of receptor cDNA plasmid and 0.5 µg of single stranded salmon sperm DNA (ssDNA, Sigma Aldrich) in PBS per 1 mL of cell suspension. Subsequently, cells were transferred to a 48-well plate coated with poly-D-lysine (7.5 x 10^4 cells/well). After incubation at 37 °C and 5% CO_2 for 48h, the medium was aspirated and 200 µL/well of fixation solution containing 4% formaldehyde (10 min, room temperature) were added. Cells were washed once with 300 µL washing buffer (150 mM NaCl, 25 mM Tris, pH 7.5) and blocked for 60 minutes (30 g · L^{-1} skim milk powder in washing buffer). After removal of the blocking solution, 200 µL/well of anti-FLAG mouse IgG (Sigma Aldrich, 1:4,000 in blocking solution) were added. After 60 min, wells were washed twice (300 µL/well) and blocked again for 60 min at room temperature, before 200 µL/well anti-mouse rabbit IgG-HPR (Sigma Aldrich, 1:20,000 in blocking solution) was added. After incubation for 60 min, cells were washed three times (washing buffer, 300 µL/well), before the substrate reaction was initiated by the addition of substrate buffer (2.8 mM o-phenylenediamine in 35 mM citric acid, 66 mM Na_2HPO_4, pH 5.0). Reactions were terminated after 45 min incubation in the dark by addition of 1 M H_2SO_4 (200 µL/well). For each well, 2 x 150 µL of the resulting mixture were transferred to a clear, flat bottom 96-well plate and absorption was measured at 492 nm in a microplate reader.

D_3R-mediated G protein activation

D_3R-mediated G protein activation was determined applying the IPOne HTRF assay (Cisbio, Codelet, France) in analogy to previous protocols.[4] In brief, HEK293T cells were transfected with 2 µg of the individual D_3R receptor constructs and 1 µg of a promiscuous $G\alpha_q$ protein,[1] using Mirus TransIT-293. 24 h post-transfection, cells were seeded into 384-well plates (black, F-bottom, Greiner Bio-One, 10,000 cells/well) in complete growth medium and allowed to adhere for 24 h at 37 °C, 5% CO_2. On the day of the assay, the medium was replaced by 10 µL/well stimulation buffer and 10 µL

of the reference agonist quinpirole (dissolved in stimulation buffer, final concentration 1 pM to 10 µM) were added. After 180 min incubation at 37 °C, the detection reagents dissolved in lysis buffer were added (IP1-d2 conjugate and anti-IP1 cryptate TB conjugate, each 5 µL/well). Incubation was continued at room temperature for 60 min, and time-resolved fluorescence resonance energy transfer was measured with a CLARIOstar microplate reader equipped with 620/10 nm and 670/10 nm emission filters. Data were normalized to the maximum (100%) and non-stimulated responses (0%) and concentration-response curves were analyzed employing the algorithms for three parameter non-linear regression implemented in PRISM6.0.

Supplementary References

1. Kostenis, E., Waelbroeck, M. & Milligan, G. Techniques: Promiscuous Gα proteins in basic research and drug discovery. *Trends Pharmacol. Sci.* **26,** 595-602 (2005).
2. Mitronova, G. Y. *et al.* New fluorinated rhodamines for optical microscopy and nanoscopy. *Chem. Eur. J.* **16,** 4477-4488 (2010).
3. Möller, D. *et al.* Functionally selective Dopamine D2, D3 receptor partial agonists. *J. Med. Chem.* **57,** 4861-4875 (2014).
4. Liu, H. *et al.* Structure-guided development of selective M3 muscarinic acetylcholine receptor antagonists. *Proc. Natl. Acad. Sci. U.S.A.* **115,** 12046-12050 (2018).
5. Tabor, A., Möller, D., Hübner, H., Kornhuber, J. & Gmeiner, P. Visualization of ligand-induced dopamine D2S and D2L receptor internalization by TIRF microscopy. *Sci. Rep.* **7,** 10894; 10.1038/s41598-017-11436-1 (2017).
